# Capacity for health economics research and practice in Jordan, Lebanon, the occupied Palestinian territories and Turkey: needs assessment and options for development

**DOI:** 10.1186/s12961-020-00586-w

**Published:** 2020-09-03

**Authors:** Adrian Gheorghe, Mohamed Gad, Sharif A. Ismail, Kalipso Chalkidou

**Affiliations:** 1 Global Health and Development, Department of Infectious Disease Epidemiology, Imperial College London, London, United Kingdom; 2 Department of Global Health and Development, London School of Hygiene & Tropical Medicine, London, United Kingdom; 3 Department of Primary Care and Public Health, Imperial College London, London, United Kingdom; 4 Center for Global Development, London, United Kingdom

**Keywords:** health economics, capacity strengthening, Eastern Mediterranean, Middle East and North Africa, online survey, bibliometric analysis

## Abstract

**Background:**

Capacity for health economics analysis and research is indispensable for evidence-informed allocations of scarce health resources; however, little is known about the experience and capacity strengthening preferences of academics and practitioners in the Eastern Mediterranean region. This study aimed to assess the needs for strengthening health economics capacity in Jordan, Lebanon, the occupied Palestinian territories and Turkey as part of the Research for Health in Conflict in the Middle East and North Africa (R4HC) project.

**Methods:**

We combined a bibliometric analysis of health economics outputs based on a literature search conducted across seven databases with an online survey of academic researchers and non-academic practitioners. The records included in the bibliometric analysis were original studies and reviews with an explicit economic outcome related to health, disease or disability, had at least one author in Jordan, Lebanon, Palestine or Turkey, and were published between January 2014 and December 2018. Two types of analyses were conducted using VOSviewer software, namely keyword co-occurrence and co-publication networks across countries and organisations. The online survey asked academic researchers, analysts and decision-makers – identified through the bibliometric analysis and regional professional networks – about previous exposure to and priorities for capacity development in health economics.

**Results:**

Of 15,185 records returned by the literature search, 566 were included in the bibliometric analysis. Organisations in Turkey contributed more than 80% of records and had the broadest and most diverse network of collaborators, nationally and internationally. Only 1% (*n* = 7) of studies were collaborations between researchers in two or more different jurisdictions. Cost analysis, cost-effectiveness analysis and health system economics were the main health economics topics across the included studies. Economic evaluations, measuring the economic burden of disease and health equity, were reported by survey respondents (*n* = 80) as the most important areas to develop in. Short courses, learn-by-doing and mentoring from an experienced professional were, in aggregate, the most preferred learning styles.

**Conclusions:**

Existing pockets of health economic expertise in the region can constitute the base of future capacity development efforts. Building confidence toward applying specific methods and trust toward stimulating cross-jurisdiction collaborations appear essential components for sustainably developing health economics capacity.

## Background

Achieving and maintaining Universal Health Coverage (UHC), whereby everyone has access to health services of adequate quality when they need them and without risk of going into poverty, depends on health systems and health decision-makers making fair, evidence-informed decisions about how to prioritise the spending of limited available resources [1]. The capacity to conduct health economics research and analyses is indispensable to achieve this end – where capacity for research can be defined as “*enhancing the abilities of individuals, organisations and systems to undertake and disseminate high quality research efficiently and effectively*” [2]. The need for such capacity could not be more relevant for health systems in the Eastern Mediterranean region, whose decision-makers are facing difficult choices in allocating scarce resources in the face of severe and context-specific constraints (e.g. conflict, political instability, stagnant economic growth) at the same time as embarking on reforms towards UHC [3]. However, the broader constraints in funding, understanding and capacity for health research ecosystems in the region have now been well documented [4, 5].

A foundational step in designing capacity development programmes is to conduct a needs assessment, understood as “*the systematic study of a problem or innovation, incorporating data and opinions from varied sources, in order to make effective decisions or recommendations about what should happen next*” [6]. Available reviews of the health economic literature conducted in the region, be they with a cross-country [7, 8] or single-country focus [9, 10], all suggest a limited use of economic evaluation and substantial scope for improving methodological quality. There are also indications that most pharmacy schools offer pharmacoeconomics courses as part of their curriculum, albeit with widely varying hours and topics [11]. However, there are many more areas of health economics other than pharmacoeconomics and economic evaluation, such as health equity or measuring the performance of service providers and health systems, both providing crucial insights in the context of reforms towards UHC. Very little is known about the capacity specific to these areas of health economics or capacity development modalities. We identified only one comprehensive needs assessment for health economics in the region, focusing on the professional development needs of junior public health professionals in Turkey [12]. In that study, health economics was the professional area (versus health policy, environmental health, medical anthropology and epidemiology) with the largest reported gap between perceived importance and self-assessed performance – particularly ‘statistical and econometric analysis’, ‘microeconomics of healthcare’ and ‘health accounting’.

We report the findings of a needs assessment for health economics research and practice in four Eastern Mediterranean jurisdictions – Jordan, Lebanon, the occupied Palestinian territories (OPT) and Turkey. The study was conducted as a preparatory step for developing a programme of health economics capacity development activities as part of the Research for Health in Conflict in the Middle East and North Africa (R4HC) project. The R4HC project aims to strengthen capacity for health research in the Middle East and North Africa region, with a focus on health, political economy of health, and complex non-communicable diseases and facilitate more effective translation of research into policy. The project has seven core partners – Hacettepe University in Turkey, American University of Beirut in Lebanon, King Hussein Cancer Center in Jordan, Birzeit University in West Bank, OPT and three United Kingdom partners (King’s College London, Imperial College London and Cambridge University).

The focus of this particular needs assessment is two-fold – (1) to describe, based on a bibliometric analysis, the recent corpus of health economics research published by authors in Jordan, Lebanon, OPT and Turkey in terms of volume, thematic areas, and collaboration patterns, and (2) to identify, using an online survey, the health economics areas that researchers and practitioners see as most important to develop as well as their preferred learning styles for health economics topics.

## Methods

Our approach combined a bibliometric analysis of health economics research outputs produced in the four target jurisdictions with an online survey of academic and non-academic health professionals. The bibliometric analysis was conducted first in order to provide an initial overview of health economics activity in terms of topics and active organisations; its main findings then informed the design and dissemination of the online survey.

### Bibliometric analysis

The bibliometric analysis focused on the question – ‘What type of health economics research has been produced in Jordan, Lebanon, OPT and Turkey between 1 January 2014 and 31 December 2018?’ There were two specific questions:

- Which health economics topics have been researched by organisations in the four jurisdictions, and to what extent?
- Which organisations have published health economics research and what are their co-publishing patterns, both within and outside the Middle East and North Africa region?

#### Data sources

Seven electronic databases were searched on 28 January 2019 – two databases with an economic focus (Econlit–Ovid and NHSEED), three health-focused databases (MEDLINE, including MEDLINE In-Process, EMBASE Classic and Embase, and Global Health – all searched through Ovid), two generalist databases (Scopus and Web of Science) and one regional database (Index Medicus for the Eastern Mediterranean Region).

#### Search strategy

A custom literature search strategy was devised with the help of an information specialist at Imperial College London Medical School Library. The search strategy comprised two types of search terms – geographic descriptors (i.e. the names of the four jurisdictions searched in the ‘institution’ field of each database) and subject descriptors (i.e. keywords pertaining to health economics). We tested four search strategies for health economics research outputs, informed by previous bibliometric reviews [13, 14, 15] and reference textbooks (details in Additional file 1), and selected a search strategy that was then adapted and ran across seven databases (full search strategies in Additional file 1).

#### Eligibility criteria

The following inclusion criteria were applied: scope – titles or abstracts that explicitly listed any economic outcome related to health, disease or disability, including but not limited to cost, cost-effectiveness, patient-reported outcomes, health expenditure, provider or health system performance, and poverty associated with accessing healthcare; geography – studies with at least one author affiliated with an organisation in Jordan, Lebanon, OPT (West Bank and Gaza) or Turkey; language – studies in any languages; publication date – studies published between 1 January 2014 and 31 December 2018; publication type – studies published as journal articles (original articles and reviews), book chapters, conference proceedings and theses. Editorials were excluded.

#### Study selection

Search results from each database were downloaded in Microsoft Excel and duplicates were removed. The following fields were retained for each record: title, abstract, authors, authors’ affiliations, publication year and publication journal/source. Titles and abstracts were screened by the lead researcher (AG). Institutional affiliation data were cleaned manually for the records meeting the inclusion criteria. For the authors’ institutional affiliation, only the highest-level affiliation was retained (e.g. Department of Internal Medicine, Faculty of Medicine, University of Buenos Aires was recorded as University of Buenos Aires).

#### Data analysis

Descriptive analyses were conducted in Microsoft Excel and bibliometric analyses were conducted using VOSviewer software version 1.6.10 [16]. Two types of analyses were conducted using VOSviewer functionalities – keyword co-occurrence and co-publication networks. Keyword co-occurrence was analysed based on VOSviewer’s built-in natural language processing algorithms, which were applied to the titles and abstracts of included records [17]. The software allows a similar analysis of author and database-indexed keywords, but a deliberate decision was made to conduct the analysis only on titles and abstracts to ensure that the body of text was as uniform as possible across sub-disciplines, journals and indexing databases as well as to minimise any systematic differences in the authors’ choice of keywords. The binary counting method was used to assess the frequency of terms, whereby a given word (sequence) is counted only once per record irrespective of how many times it appears within it.

For co-publication networks, both organisations and jurisdictions were analysed based on the authors’ affiliations. When there were more authors from a single organisation, the organisation was counted only once. The fractional counting method was used to ascertain country and organisation authorship, whereby the strength of a co-authorship link between two organisations is determined not only by the number of records co-authored but also by the total number of organisations of each of record – this method reduces the influence of large collaborations. For example, if a study is authored by authors from five different organisations, each link between two co-authoring organisations gets a strength of 1/5 (versus a strength of 1 if the full counting method had been employed) [18].

Results are presented as network visualisations, where each circle depicts a keyword (or institution); the size of the circle is proportional with the number of records mentioning that keyword (or authored by someone at that institution). The closer two keywords (institutions) appear and the thicker the links between them and the larger the number of records in which they appear (co-author) together. The colours distinguish between clusters of keywords (institutions), i.e. collections of keywords (institutions) that tend to appear (publish) together. A keyword (institution) can only belong to one cluster.

### Online survey

The survey focused on the following question: ‘What are the capacity development priorities for health economics among academic and non-academic professionals in Jordan, Lebanon, OPT and Turkey?’ There were three specific questions:

- Which health economics topics have respondents received training in and applied in research or analysis, respectively, over the past 5 years?
- Which health economics topics are respondents most interested to develop in the future?
- What are the respondents’ preferred modalities to develop their health economics skills and knowledge?

#### Survey sample

Survey respondents were identified through several means. Firstly, the email addresses of the authors of the 566 records included in the bibliometric analysis were identified based on information publicly available online on their respective institutions’ and departments’ websites. Secondly, R4HC project partners were consulted on the relevant academic and non-academic institutions/departments to approach in each country. Thirdly, representatives of relevant regional organisations (e.g. WHO Office for the Eastern Mediterranean Region) and in-country professional associations (e.g. local chapters of the International Society for Pharmacoeconomics and Outcomes Research (ISPOR)) were approached for additional relevant professionals/organisations and for support with disseminating the questionnaire within their networks. A consolidated list of potential respondents was created by combining these three sources. Only researchers affiliated with university departments of public health, pharmacy, health economics, health policy and economics were retained as these departments were judged to be likely hosts of institutional health economics expertise.

#### Recruitment

Individuals on the consolidated recruitment list were sent an email invitation to participate in the survey; the recruitment email explained the purpose of the study and invited recipients to participate by clicking the enclosed weblink, which lead to the online questionnaire. The recruitment email also encouraged recipients to forward the recruitment email to colleagues for whom health economics is a discipline relevant to their professional role. A total of 286 unique individuals across the four target jurisdictions and the region were sent the invitation email from 9 September 2019. A single reminder email was sent to all recipients around 20 September 2019.

#### Survey instrument

The survey instrument (Additional file 1) distinguished between two tracks based on the respondents’ professional role, as reported in the first question. The technical track applied to respondents self-identifying as academic researchers, technical analysts (non-academic) and clinicians); the 14 questions focused on detailed health economics methods. The managerial track applied to respondents self-identifying as policy managers or healthcare administrators; the 12 questions focused on the application of health economics methods to answer higher-level policy questions. In questions about past exposure to health economics topics, respondents were presented with multiple choice items, e.g. ‘no exposure to the topic’, ‘I have worked on this topic’ or ‘I am an expert in this topic’. In questions concerned with future priorities in terms of health economics capacity development, respondents were asked to rank the items (using the drag and drop functionality of the survey platform) from the most important to the least important.

This approach was preferred to the Likert-scale rating approach used by the WHO Hennessy-Hicks needs assessment tool [19], which compares, for a given skill, the self-reported achievement and the perceived importance of the skill for the professional role, for two reasons, namely health economics is a niche discipline in the public health space and preliminary discussions with R4HC consortium partners and other academics in Jordan, Lebanon and Palestine indicated that there were very few ‘pure’ health economists in these settings. As such, a deliberate decision was made to broaden the professional profiles of the response sample while acknowledging that their professional role specifications (where these are available) may not include explicit health economics competencies.

The draft survey (English version) was piloted with five researchers (including two health economists) in the Global Health Development group at Imperial College London and five health systems researchers with regional experience (including four health economists), following which modifications were made based on their feedback. The English version was then translated into Arabic and Turkish by native speaker health economists. Based on the pilot we anticipated that completing the survey would take between 10 and 15 minutes; this information was included in the participant information sheet linked to the recruitment email ‘[the survey] should take about 10–12 minutes to complete’. No incentives were offered to respondents for completing the survey.

#### Data collection

Survey responses were collected using the Qualtrics platform through the Principal Investigator’s Imperial College’s secure account. The online questionnaire was open for a period of 5 weeks (9 September to 14 October 2019), following which it was locked and responses were no longer accepted. Each page of the online questionnaire allowed responses to be collected in English, Turkish or Arabic based on the translated versions, as explained above.

#### Data analysis

Survey responses were downloaded in spreadsheet format for cleaning, following which they were transferred to R statistical software for analysis [20]. Partial responses were kept on the Qualtrics platform, but not downloaded for analysis. Responses to each question were summarised using appropriate descriptive statistics (e.g. proportions) and by subgroups (e.g. by country, by type of institution).

## Results

### Summary of studies included in the bibliometric analysis

**Fig. 1:**
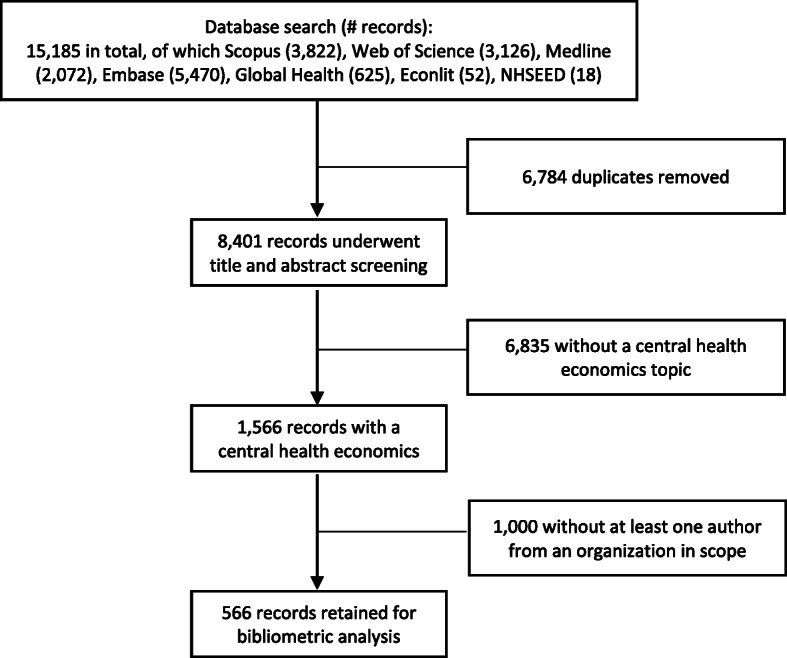
Study inclusion flowchart

The literature search identified 15,185 records across all databases, of which 8401 were unique records whose titles and abstracts were screened against the inclusion criteria (Fig. 1). Of the 1566 records with a central health economics topic, 566 had at least one author from an institution in either Jordan, Lebanon, OPT or Turkey; these 566 articles were retained for the bibliometric analysis.

Most included records were journal articles (*n* = 461, 81%); there were also 102 conference proceedings (18%, most of them at ISPOR European or ISPOR World congresses) and 3 books/book chapters (Table 1). About half had authors from one or two distinct organisations, but there were also large collaborations between five or more organisations (*n* = 121, 21%). Of the collaborations between two or more organisations, half were international collaborations and half were collaborations among domestic organisations only.

**Table 1:** Summary of studies included in the bibliometric analysis (*n* = 566)

Characteristic	***N***	%
Publication year		
2014	101	17.8
2015	117	20.7
2016	121	21.4
2017	111	19.6
2018	116	20.5
Total	566	100.0
At least one author from an organisation in		
Jordan	38	6.7
Lebanon	53	9.4
Occupied Palestinian Territories	14	2.5
Turkey	464	82.0
Any two of the above	7	1.2
Any three from the above	0	0
Number of distinct organisations per record		
One	160	28.3
Two	133	23.5
Three	96	17.0
Four	56	9.9
Five or more	121	21.4
Total	566	100.0
Of collaborations between at least two organisations		
International collaborations	201	49.5
Domestic-only collaborations	205	50.5
Subtotal	406	100.0
Publication type		
Journal article	461	81.4
Conference proceeding	102	18.0
Book or book chapter	3	0.5
Total	566	100.0
Type of organisation		
Academic (e.g. university, research institute)	506	89.4%
Service provider (e.g. teaching hospital)	162	28.6%
Public administration (e.g. Ministry of Health)	53	9.4%
Industry (e.g. pharma company, consultancy)	129	22.8%
Other (e.g. Non-governmental organisation, international organisation)	11	1.9%
Top journals (by number of articles)		
*Value in Health*	70	15.1
*PLoS One*	7	1.5
*International Journal of Health Planning and Management*, *Value in Health Regional Issues*	6	1.3
*The Lancet*, *Turkish Journal of Medical Sciences*, *Tuberkulos ve Toraks*	5	1.1

Most records had at least one author from a Turkish organisation (*n* = 464, 82%), followed by Lebanon (*n* = 53), Jordan (*n* = 38) and OPT (*n* = 15). Only 7 records (1% of total) were the product of collaborations between authors in at least two of the four jurisdictions: Jordan–Lebanon (2), Jordan–Turkey (1) and Turkey–OPT (4); there was no record with authors in three or all four jurisdictions. Most records had authors affiliated with an academic organisation (*n* = 506, 89%), but there was also authorship from service providers (e.g. teaching hospitals) and healthcare industry organisations (29% and 22%, respectively).

*Value in Health* was by far the most common source of journal articles (*n* = 70, 15%), with the other top ten journals by number of records representing a combination of international (e.g. *The Lancet*, *International Journal of Health Policy and Management*) and national journals (e.g. *Turkish Journal of Medical Sciences*, *Jordan Journal of Pharmaceutical Sciences*). Further disaggregations by country in terms of collaboration patterns and research productivity are presented in Additional file 1.

### Bibliometric analysis – health economics topics

**Fig. 2:**
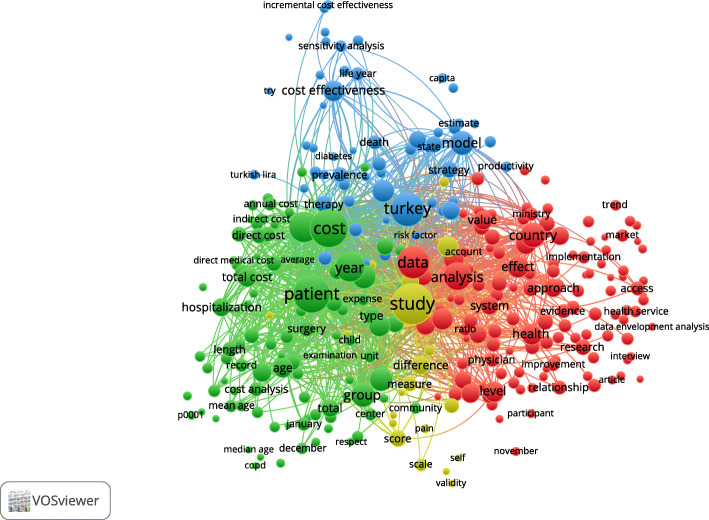
Text co-occurrence patterns in titles and abstracts, all included records (*n* = 566). Notes: only countries appearing in at least 5 records are displayed; minimum 10 topics per cluster

Three health economics topics are apparent across the included records, namely cost analysis, cost-effectiveness analysis and health system economics (Fig. 2). Cost analysis (green cluster) is illustrated by terms like ‘total cost’, ‘hospitalisation’, ‘economic burden’ and ‘annual cost’. This area is closely linked to cost-effectiveness analysis (blue cluster), which contains terms like ‘cost-effectiveness’, ‘sensitivity analysis’ and ‘life year’. Health system economics (red cluster) is the richest category and includes a large and varied body of terms, e.g. ‘country’, ‘system’, ‘efficiency’, ‘analysis’, ‘implementation’, ‘access’ and ‘income’.

Further results of the text analysis, by type of publication (conference proceedings versus journal articles) and by jurisdiction, are provided in Additional file 1. From a disease burden perspective, no topics immediately stood out. However, most keywords pointed towards chronic conditions, particularly in Lebanon (e.g. ‘tobacco’, ‘cancer’, ‘stroke’). From a service delivery perspective, ‘pharmaceuticals’ and ‘hospital care’ were invoked the most frequently.

### Bibliometric analysis – co-publication patterns

Turkish organisations co-published across a network of 55 different countries, while OPT and Lebanon had fewer links (36 and 30 different countries, respectively) and Jordanian organisations had links only to 15 countries (Additional file 1, Fig. A1.1). Notably, Turkey, Lebanon and OPT appear so close to each other in the visualisation not because authors from these jurisdictions publish together, but because they tend to publish with authors in the same third-party countries – most prominently United States and, for Turkey and Lebanon, also countries like Canada and France.

### Online survey respondents’ profile

Of 286 email invitations sent, 161 individuals opened the survey and 83 responded to all questions (51.6% completion rate). Three complete responses were removed as the respondents now worked outside the four target jurisdictions (in France, Switzerland and United Arab Emirates), leaving 80 complete responses in the analysis. The characteristics of the 80 respondents are summarised in Table 2. The majority of respondents self-identified as academic researchers and had completed a doctoral degree, yet had varying levels of experience as reflected by the time since highest educational attainment.

**Table 2:** Summary of survey respondents’ profile (*n* = 83)

Question	***N***
**Which country do you work in?**	
France	1
Jordan	16
Lebanon	13
Occupied Palestinian Territories	33
Switzerland	1
Turkey	18
United Arab Emirates	1
**Which of the following best describes your current professional role?**	
Academic research	56
Clinical activity, for example, as a practicing physician or pharmacist	9
Healthcare management, for example, as a hospital administrator	4
Policy management, for example, as a head of unit in a government agency	3
Technical expert (non-academic), for example, as a health financing specialist	2
Other	9
**Highest educational attainment**	
Doctorate (for example, PhD, DrPH)	50
Master (for example, MA, MSc, MPH)	23
Undergraduate degree (for example, Medicine, Dentistry, Economics)	7
Other	3
**Years since highest educational attainment**	
Less than 5 years	38
Between 5 years and 10 years	16
More than 10 years	29
**Type of employer**	
Government agency	6
Healthcare provider, for example, a hospital	9
International organisation	2
Non-governmental organisation	4
Private sector, for example, consultancy or pharmaceutical industry	4
University or research institute	54
Other	4

### Online survey – health economics topics

The proportions of respondents reporting no previous exposure to the listed health economics topics were between 35% and 65%, depending on the topic (Table 3). ‘Economic evaluation’ and ‘measuring health utilities and health-related quality of life’ were the topics that most respondents reported to be familiar with but, even for these, less than 40% of respondents reported to have worked on them directly. At the other end of the spectrum, the least previous exposure across the sample (about 15% of respondents) was reported for ‘political economy analysis’, ‘measuring health equity’ and ‘measuring the efficiency of health systems or providers’. Respondents who provided free text details on their practical experience mentioned the difficulty of obtaining cost-specific data and navigating bureaucracy as main challenges of applying these methods in their context.

**Table 3:** Previous exposure to and importance of future development in health economics topics among survey respondents (*n* = 73)

Health economics topic	Previous exposure - # respondents (%)					Importance of future development	
	I am an expert on this topic	I completed a face-to-face course on this topic	I completed an online course on this topic	I have worked on this topic	No exposure to this topic	Average rank (SD, IQR)	# respondents ranking the topic in top 3 (%)
Measuring the economic burden of disease	9 (12.3%)	14 (19.2%)	4 (5.5%)	20 (27.4%)	33 (45.2%)	4.56 (2.67, 3-6)	32 (43.8%)
Economic evaluation	10 (13.7%)	14 (19.2%)	5 (6.8%)	28 (38.4%)	28 (38.4%)	4.68 (3.06, 2-7)	35 (47.9%)
Measuring health equity	5 (6.8%)	14 (19.2%)	1 (1.4%)	12 (16.4%)	48 (65.8%)	4.96 (2.76, 3-7)	25 (34.2%)
Measuring health utilities and HRQoL	10 (13.7%)	14 (19.2%)	4 (5.5%)	30 (41.1%)	26 (35.6%)	5.18 (2.99, 3-8)	25 (34.2%)
Measuring the efficiency of health systems or providers	6 (8.2%)	11 (15.1%)	3 (4.1%)	10 (13.7%)	47 (64.4%)	5.27 (2.83, 3-7)	24 (32.9%)
Measuring healthcare costs	8 (11.0%)	14 (19.2%)	2 (2.7%)	23 (31.5%)	35 (47.9%)	5.33 (2.53, 4-7)	18 (24.7%)
Formal policy analysis	3 (4.1%)	10 (13.7%)	1 (1.4%)	16 (21.9%)	44 (60.3%)	5.96 (2.72, 4-8)	16 (21.9%)
Measuring preferences of health workers or patients	8 (11.0%)	10 (13.7%)	2 (2.7%)	16 (21.9%)	41 (56.2%)	6.05 (2.70, 4-8)	17 (23.3%)
Political economy analysis	3 (4.1%)	9 (12.3%)	2 (2.7%)	15 (20.5%)	48 (65.8%)	6.71 (3.4, 4-10)	17 (23.3%)
Quasi-experimental methods	8 (11.0%)	12 (16.4%)	2 (2.7%)	14 (19.2%)	40 (54.8%)	7.15 (3.0, 5-10)	10 (13.7%)
Other	-	-	-	-	-	10.14 (1.7, 10-11)	0 (0%)

For “Previous exposure” the survey question was “Thinking about the past 5 years (since September 2014), choose the statements that apply to you for each of the following topics. Choose all statements that apply. For the purpose of this question, the following are NOT sufficient to be considered a 'course': one-day activities; individual study without an instructor; attending one-off lectures, conference presentations, seminars, webinars.”; line totals do not add up to 100% because respondents could choose more than one response item under each health economics topic

For “Importance of future development” the survey question was “Which of the following health economics topics do you consider most important for you to develop in the future?

Order (drag and drop) the following options from the most important (1) to the least important (11).” Results exclude three respondents outside the four focal jurisdictions and seven respondents with policy or management roles

*Abbreviations*: *HRQoL* health-related quality of life, *IQR* interquartile range, *SD* standard deviation

‘Measuring the economic burden of disease’, ‘economic evaluation’, ‘measuring utilities and health-related quality of life’ and ‘measuring health equity’ were, on average, the topics that respondents reported as most important for them to develop in the future, while ‘measuring preferences’, ‘political economy analysis’ and ‘quasi-experimental methods’ were, on average, the least important for their future professional development. Results were consistent across countries and types of professional roles, with only minor differences among them, e.g. ‘measuring the efficiency of health systems and providers’ was among top five topics in Lebanon but not in the other three jurisdictions (Additional file 1). Survey respondents on the managerial track (*n* = 7) ranked ‘tools for navigating the politics of health sector reform’ as their top professional development priority, ahead of more technical topics like ‘costing health benefit packages’.

### Online survey – capacity development modalities

Respondents ranked short courses (2–5 days), learn-by-doing and mentoring from an experienced professional highest in terms of preferred learning styles for the health economics topics they perceived as most important for their future professional development (Table 4). As with health economics topics for future development, preferences were generally consistent across countries and types of professional roles, also with few differences, e.g. stronger preference for online courses with a certificate of completion in Lebanon versus the other three jurisdictions.

**Table 4:** Preferred learning style for health economics topics among survey respondents (*n* = 80)

	Average rank (SD, IQR)	Number of respondents ranking the option in top 3 (%)
Face-to-face course (2–5 days)	2.80 (2.06, 1–4)	58 (72.5%)
Direct mentoring from an experienced professional	3.61 (1.74, 2–5)	43 (53.8%)
Learn by doing	3.67 (2.20, 2–5.25)	43 (53.8%)
Master-level course in academic institution	4.47 (2.18, 3.6)	27 (33.8%)
Peer-to-peer learning	4.51 (1.81, 3–6)	25 (31.3%)
Online course with graded assessment(s) and certificate of completion	4.68 (1.91, 4–6)	19 (23.8%)
Online course without graded assessment(s)	5.21 (1.95, 3.75–7)	20 (25%)
Other	7.02 (1.88, 7–8)	5 (6.3%)

The survey question was ‘For the top 3 [health economics] topics you ranked above, what would be your preferred learning style? Order (drag and drop) the following options from the most preferred (1) to the least preferred (8)’. Excludes three respondents outside the four focal jurisdictions

*IQR* interquartile range, *SD* standard deviation

## Discussion

### Summary of findings

The bibliometric analysis found that recent health economics research in Jordan, Lebanon, OPT and Turkey appears concentrated on a limited number of themes, namely cost analysis and cost-effectiveness analysis. There appears to be a shared thematic focus across the four jurisdictions on pharmaceuticals, hospital care and chronic disease. Only 1% (*n* = 7) of the included research outputs were the product of cross-jurisdiction collaborations between researchers in at least two of the four jurisdictions included in this study.

Economic evaluation and economic burden of disease were ranked, on average, highest in terms of importance for future development. Short courses, learning by doing and mentoring from an experienced professional were, on average, the learning styles ranked highest by way of preference for developing these topics. Findings were generally consistent across countries and types of professional role.

### Interpretation of findings

The combined findings of the bibliometric analysis and the online survey indicate that some health economics research capacity appears to be in place in the focal jurisdictions, particularly for economic evaluation, measuring health utilities and healthcare costs. The focus of research outputs on pharmaceutical technologies, the dominance of conference presentations (predominantly ISPOR) and of *Value in Health* as the target journal (particularly for Turkish authors), combined with the still nascent processes for incorporating economic evidence into government healthcare planning and decision-making (particularly in Jordan, Lebanon and OPT), suggest that the pharmaceutical industry may have had an important role in shaping the focus of existing health economics capacity in the region.

By contrast, there has been much less recent research and exposure to methods relating to the analysis of healthcare markets (e.g. competition, insurance, provider and patient behaviour), equity (in health outcomes, access to health services or health financing), or the determinants of provider performance. These three topics are particularly under-represented in research from organisations in Lebanon and Jordan.

The differences among the four target jurisdictions may well explain some of these thematic variations. Turkey stands out in terms of the volume and thematic breadth of research outputs, potentially signalling a stronger, more mature health economics research base – a finding aligned with the size of its economy and of its higher education sector as well as more than a decade of structural health sector reforms [21]. On the other hand, the constraints related to financing, information systems, governance and security, among others, inherent to health sectors in Jordan, Lebanon and OPT have been comparatively more severe over the same time interval and it is conceivable that the opportunities for health economics research and capacity-building have been correspondingly fewer and less diverse.

Our results must be interpreted with caution. Firstly, analysing research outputs and individual professionals’ views of capacity development does not tell the whole story of research capacity. The existence of health economics research outputs is not necessarily equivalent to capacity being in place and the absence of research outputs does not necessarily mean that research is not being conducted or that research capacity has never been in place. For example, researchers at Birzeit University published original research on health equity in 2008–2009 [22, 23] but this individual capacity is no longer in place at Birzeit University.

Secondly, some thematic areas (e.g. economic evaluation) being prioritised more highly by survey respondents in terms of their future professional development relative to others (e.g. provider performance analysis, political economy analysis) does not necessarily mean that the latter topics are perceived as less valuable, necessary or important in a given context. It may well be the case that weak policy demand for certain types of analyses, lack of career progression opportunities or the structure of existing public health/health systems/health economics training are prominent considerations shaping the responses.

The low share of cross-jurisdiction collaborations in health economics research (*n* = 7 of 566 records) is striking. Turkish–Arabic language barriers are unlikely to be significant as five of the seven collaborative pieces included a Turkish institution – also despite the share of entirely domestic collaborative publications being the highest in Turkey among the four jurisdictions. The availability and accessibility of regional funding pools for health research requires further attention as it is likely to stimulate long-term cross-jurisdiction collaborations and multiply opportunities for capacity development. The progress made to this effect in Latin America can offer potentially useful insights [24].

### Strengths and limitations

To our knowledge, this is the first systematic exploration of capacity for health economics research in the Eastern Mediterranean region that goes beyond economic evaluation. The combination of a comprehensive literature search, bibliometric analysis and online survey generated consistent and complementary results.

There are also limitations. We captured capacity primarily among knowledge producers (i.e. academics and technical analysts), which are only one category of stakeholders in the healthcare priority-setting landscape for a given context [25]. Furthermore, we focused on individual-level capacity and not on organisational or institutional levels in the absence of established health economics academic departments in these jurisdictions. In the bibliometric analysis, ascertaining whether an article had a central health economics topic based on single-screening the title and abstract entailed a degree of subjectivity, for example, it was particularly difficult to adjudicate articles about ‘access to health services’ as it was difficult to distinguish between health services research and health equity as the central topic. Assessing the methodological quality of the health economic literature was left outside the scope of the analysis given the expected large number of records and the broad conceptual scope – quality criteria are available for various types of economic analysis, such as economic evaluation [26] and using data envelopment analysis to measure the performance of service providers [27], but these would be difficult to reconcile across study types.

The survey sample was not statistically representative, the response rate was at most 30% (most likely lower given the snowball dissemination of the recruitment email) and the number of responses per country was of around 20 – as such, results can offer only an indication of the true picture. However, the completion rate of 50% was in line with acceptable ranges for unsolicited online surveys. Furthermore, no exhaustive definitions of response items were provided (e.g. health equity analysis, political economy analysis); therefore, it is possible that responses over- or under-estimate the exposure or perceived importance for future development if respondents did not fully recognise the terms. The low response rate among policy-makers and health administrators precludes any meaningful conclusion for this category of respondents.

Only 21 abstracts (4% of total) included at least one of the terms ‘refugee’, ‘conflict’ or ‘Syria’ – somehow surprising given the protracted tensions in OPT and the proximity of the Syrian conflict (started early 2012) during the time scope of this review. Given our inclusion criteria stipulating at least one author to be affiliated with an institution in the four jurisdictions, we may have missed outputs from international organisations focusing on this topic. At the same time, it is also possible that health economics research conducted by domestic authors tends to focus rather on resident populations than on displaced populations, in line with the local conceptualisation of what UHC means; however, a more comprehensive and targeted analysis would be better placed to ascertain whether this is actually the case.

### Implications for capacity development

Using these findings to inform capacity development activities with a health economics focus requires attention from the outset to the various levels at which capacity needs developing in a given context, the types of capacity to be built at each level and, above all, the importance of being clear about the purpose for capacity development in the first place as these will determine the necessary activities. For instance, there are indications of health research waste in Palestine [28] in the absence of research priority-setting mechanisms [29]; in this case, developing technical expertise can only bring limited results if investment is not directed also towards overcoming barriers to making research more relevant for policy-making, particularly as there is evidence of little engagement in knowledge translation activities from health research institutions in the region [30]. The influence of conflict adds another dimension relevant to the region as a whole, as challenges to developing capacity are more taxing compared to ‘conventional’ settings and corresponding capacity development activities require, among others, focusing on organisations and functions (rather than individuals), increasing trust within and across research teams and research participants, and considering novel ways to conduct and communicate research results timely [31].

Our findings generate several working directions for a capacity development plan. First, making the most of already available health economics capacity appears both necessary and possible. Two academic centres appear to be at the centre of the co-publication networks in their respective countries – Hacettepe University (Turkey) and the American University of Beirut (Lebanon) – and they are therefore immediate candidates for co-designing and co-delivering capacity development activities to other national partners. Second, capacity development activities that focus on both knowledge transfer (e.g. short courses), practical applications (e.g. learn by doing) and confidence building (e.g. mentoring) appear to respond best to the potential beneficiaries’ expectations; an additional element of activities aimed at building trust and collaboration routines across institutions (e.g. regional visiting fellowships) could also energise cross-jurisdiction collaborations. Third, linking together existing pockets of expertise within and across countries appears sensible with a view to sustainability, for example, by leveraging the existing EMRO-DCP Health Economic Evaluation Network [32] and existing academic concentrators of expertise toward establishing a regional centre of excellence for health economics research. Fourth, stimulating links between policy-makers and researchers requires particular attention with a view to encouraging locally led research studies to be commissioned by decision-makers seeking answers to pressing questions on the path towards UHC – it is unlikely that health economics capacity will be sustainably built in the absence of strong and sustained policy demand for such evidence.

## Conclusions

Future efforts to strengthen health economics capacity can build on the existing available expertise as a starting point for developing tailored programmes that take into account the specificities of each jurisdiction, reflected, for example, in preferences for learning styles and in the nature of existing professional networks. Developing confidence, trust and effective collaborations, both among existing pockets of expertise and between researchers and policy-makers, are potentially promising directions for the design of future capacity development programmes.

## Supplementary information

**Additional file 1.** Supplementary information.

## Data Availability

The datasets used and/or analysed during the current study are available from the corresponding author on reasonable request.

## Abbreviations

ISPOR: International Society for Pharmacoeconomics and Outcomes Research
OPT: Occupied Palestinian Territories
R4HC: Research for Health in Conflict in the Middle East and North Africa project
UHC: Universal Health Coverage
