# Supplementary material for: Capacity for health economics research and practice in Jordan, Lebanon, the occupied Palestinian territories and Turkey: needs assessment and options for development
Source: Health Res Policy Syst. 2020 Sep 3;18:99. doi: 10.1186/s12961-020-00586-w (PMC7469424; doi:10.1186/s12961-020-00586-w)

Appendix 1. Details of bibliometric analysis methods and findings

This section describes the process for selecting the search strategy that informed the bibliometric analysis, as well as the detailed findings of the bibliometric analysis itself. The actual search strategies are presented in full in Appendix 2.

**Selecting the search strategy**

The first step was to devise a custom search strategy for health economics. The tables of contents of four reference health economics textbooks were extracted: Handbook of Health Economics (vol 1A and 1B, 2000, edited by Culyer AJ and Newhouse JP; vol 2, 2011, edited by Pauly MV, Mcguire TG and Barros PP), Oxford Handbook of Health Economics (2011; Glied S and Smith PC eds), Elgar Companion to Health Economics (2006, edited by Jones AM), and Health Economics (2006, edited by Zweifel P, Breyer F and Kifmann M). The titles of all individual chapters were analysed thematically, resulting in the distillation of five thematic areas: health expenditure and financing; health care markets (including health insurance); equity; measuring health and evaluating health technologies; and measuring performance (Table A1.1). The concepts associated with each thematic area informed the formulation of the literature search terms.

Table A1.1 Thematic areas used to operationalise the “*health economics*” literature search strategy

| **Thematic area** | **Concepts** |
| --- | --- |
| Health expenditure and health financing | Expenditure; financing; cost; budget; economic burden; cost of illness |
| Health care markets (including insurance) | Capitation; fees; reimbursement; purchasing; contracting; competition; incentives; provider and consumer behaviour; health insurance |
| Equity | (in)equity; (in)equality; catastrophic/impoverishing/out-of-pocket expenditure |
| Measuring health and evaluating health technologies | Economic evaluation; pharmacoeconomics; cost; cost-effectiveness; cost-utility; cost-benefit; QALY; DALY; patient-reported outcomes; utility values |
| Measuring performance | Efficiency; productivity; performance – of health systems and service providers |

Notes: synonyms and related concepts were used to operationalize the listed concepts into literature search items, please see Appendices 1-2 for further details.

In the second step, the performance of several search strategies was assessed to inform the selection of the search strategy to be employed in the analysis. This step was deemed necessary for balancing comprehensiveness with keeping the review process manageable, in anticipation of the large number of records that would be returned by a generic strategy. The performance of each search strategy was evaluated in terms of two parameters: specificity, measured as the proportion (%) of records with a central health economics topic from the total number of records returned by each search strategy; and sensitivity, measured as the proportion (%) of records with a central health economics topic returned by each strategy from the total number of distinct health economics articles identified by all strategies combined (Pitt, Goodman and Hanson 2016).

The four tested search strategies were constructed to balance sensitivity and specificity to various degrees – full details are below. In increasing order of expected sensitivity (decreasing order of expected specificity):

- #1 JakovljevicTI: the search strategy used by (Jakovljevic and Pejcic 2017), which searches only article TITLES
- #2 customTI: a strategy based on (Jakovljevic and Pejcic 2017), with additional search terms; also restricted to article TITLES
- #3 customTIABKEY_proximity7: a custom strategy informed by Table A1.1, searching both TITLES, ABSTRACTS and KEYWORDS with proximity operator, i.e. health-related and economic-related search terms at no more than 7 words apart. The level of proximity (7 words) was chosen in consultation with the information specialist.
- #4 customTIABKEY: a custom search strategy informed by Table A1.1, searching TITLES, ABSTRACTS and KEYWORDS without a proximity operator between health-related and economic-related search terms.

The four search strategies were tested by extracting and manually screening Scopus and MEDLINE (Ovid) indexed articles published in 2018. The search strategy minimizing the number of returned articles and maximizing sensitivity and specificity would be carried forward. In the third and final step, the selected search strategy would be adapted to and implemented for all other five databases.

Of the four tested literature strategies (details below), strategies #1 and #2 had the highest specificity levels but were discarded due to their sensitivity below 60%, which risked omitting about half the relevant articles (Table A1.2). Strategy #3 (custom TITLE and ABSTRACT with proximity operator) was carried forward as it provided the best balance of sensitivity and specificity: it returned 2-3 times fewer results than the most sensitive strategy (#4) for comparable sensitivity. The final search strategies for all databases are in Appendix 2.

Table A1.2 Results of tested search strategies for “*health economics*”

| **Scopus (2018)** |  |  |  |  |
| --- | --- | --- | --- | --- |
| **Search strategy** | **total** | **h econ** | **% sens** | **% spec** |
| #1 JakovljevicTI | 146 | 36 | 41% | 25% |
| #2 customTI | 157 | 47 | 54% | 30% |
| #3 customTIABKEY proximity7 | 840 | 80 | 92% | 10% |
| #4 customTIABKEY | 2,474 | 80 | 92% | 3% |
| TOTAL | 3,617 | 87 |  |  |
| **MEDLINE (2018)** |  |  |  |  |
| **Search strategy** | **total** | **h econ** | **% sens** | **% spec** |
| #1 JakovljevicTI | 38 | 28 | 54% | 74% |
| #2 customTI | 68 | 31 | 60% | 46% |
| #3 customTIABKEY proximity7 | 295 | 49 | 94% | 17% |
| #4 customTIABKEY | 793 | 52 | 100% | 7% |
| TOTAL | 1,194 | 52 |  |  |

Notes: “total” denotes the number of records retrieved. “h econ” denotes the number of records with a central health economics topic. “% sens” is calculated as the ratio of “h econ” and “total”. “% spec” is calculated as the ratio of the row “h econ” and the column maximum “h econ”.

Ovid MEDLINE(R) and Epub Ahead of Print, In-Process & Other Non-Indexed Citations, Daily and Versions(R) 1946 to January 23, 2019

| # | Strategy | Hits |
| --- | --- | --- |
|  | **customTIABKEY** |  |
| 1 | (expenditure* or expense* or fund* or budget* or spend* or price* or financ* or cost* or economic* or macroeconomic* or pharmacoeconomic* or affordab* or purchas* or reimburse* or pre-pay* or co-pay* or copay* or insurance or market* or "cost-effective*" or "cost-utility" or "cost-benefit" or "economic evaluation" or "cost per death" or "cost per case" or "cost per infection" or "cost per life" or "cost per disability-adjusted" or "cost per quality-adjusted" or "cost per qaly" or qaly or daly or "technology assessment" or performance or productiv* or efficien* or "benefit-incidence" or "data envelopment" or "stochastic frontier" or malmquist or "Pabon-Lasso").mp. | 3188272 |
| 2 | (health or medical or medicine* or medication or disease* or illness or treatment or therap* or pharmaceutical* or chemotherap or prevention or prescription* or drug* or pharmaceutical* or inpatient or outpatient or evaluation or surgery or surgical or immuniz* or immunis* or screening or hospital* or physician* or utility or utilities or "discrete choice" or "contingent valuation" or "patient-reported" or prom).mp. | 17749583 |
| 3 | (turkey or jordan or leban* or palestin* or gaza or "West Bank").in. | 256487 |
| 4 | 1 and 2 and 3 | 19718 |
| 5 | limit 4 to (humans and yr="2018") | 793 |
|  | **customTIABKEY_proximity7** |  |
| 6 | ((expenditure* or expense* or fund* or budget* or spend* or price* or financ* or cost* or economic* or macroeconomic* or pharmacoeconomic* or affordab* or purchas* or reimburse* or pre-pay* or co-pay* or copay* or insurance or market* or "cost-effective*" or "cost-utility" or "cost-benefit" or "economic evaluation" or "cost per death" or "cost per case" or "cost per infection" or "cost per life" or "cost per disability-adjusted" or "cost per quality-adjusted" or "cost per qaly" or qaly or daly or "technology assessment" or performance or productiv* or efficien* or "benefit-incidence" or "data envelopment" or "stochastic frontier" or malmquist or "Pabon-Lasso") adj7 (health or medical or medicine* or medication or disease* or illness or treatment or therap* or pharmaceutical* or chemotherap or prevention or prescription* or drug* or pharmaceutical* or inpatient or outpatient or evaluation or surgery or surgical or immuniz* or immunis* or screening or hospital* or physician* or utility or utilities or "discrete choice" or "contingent valuation" or "patient-reported" or prom)).mp. | 767859 |
| 7 | 3 and 6 | 6234 |
| 8 | limit 7 to (humans and yr="2018") | 295 |
|  | **customTI** |  |
| 9 | ((expenditure* or expense* or fund* or budget* or spend* or price* or financ* or cost* or economic* or macroeconomic* or pharmacoeconomic* or affordab* or purchas* or reimburse* or pre-pay* or co-pay* or copay* or insurance or market* or "cost-effective*" or "cost-utility" or "cost-benefit" or "economic evaluation" or "cost per death" or "cost per case" or "cost per infection" or "cost per life" or "cost per disability-adjusted" or "cost per quality-adjusted" or "cost per qaly" or qaly or daly or "technology assessment" or performance or productiv* or efficien* or "benefit-incidence" or "data envelopment" or "stochastic frontier" or malmquist or "Pabon-Lasso") and (health or medical or medicine* or medication or disease* or illness or treatment or therap* or pharmaceutical* or chemotherap or prevention or prescription* or drug* or pharmaceutical* or inpatient or outpatient or evaluation or surgery or surgical or immuniz* or immunis* or screening or hospital* or physician* or utility or utilities or "discrete choice" or "contingent valuation" or "patient-reported" or prom)).ti. | 153145 |
| 10 | 3 and 9 | 1119 |
| 11 | limit 10 to (humans and yr="2018") | 68 |
|  | **JakovljevicTI** |  |
| 12 | (economic or economics or economy or economical* or pharmacoeconomic* or cost or costs or expenditure or expenditures or budget or expense or expenses or market or spend or spends or spending or payment or payments or out-of-pocket).ti. | 183400 |
| 13 | (burden or disease or diseases or care or driver or drivers or treatment or treatments or treating or treated or treat or management or pattern or patterns or inpatient or outpatient or hospital or medical or insurance or out-of-pocket or service or services or drug or drugs or pharmaceutical or pharmaceuticals or resource or resources or therapy or coverage or health or healthcare or health-care or diagnosis or diagnostic or medicine or medicines or medication or prevention or vaccination or vaccine or screening or screen or analysis or vaccine or utilization or allocation or illness or illnesses or evaluation or impact or saving or savings or containment or prophylaxis or surgery or rehabilitation or generic or generics or biosimilar or biosimilars or injection or injections or implant or implants or implantation or psychiatry or technology or assessment).ti. | 7550085 |
| 14 | 3 and 12 and 13 | 727 |
| 15 | limit 14 to (humans and yr="2018") | 38 |

Scopus

| Strategy | Search | Hits |
| --- | --- | --- |
| JakovljevicTI | TITLE ( economic OR economics OR economy OR economical* OR pharmacoeconomic* OR cost OR costs OR expenditure OR expenditures OR budget OR expense OR expenses OR market OR spend OR spends OR spending OR payment OR payments OR "out-of-pocket" ) AND TITLE ( burden OR disease OR diseases OR care OR driver OR drivers OR treatment OR treatments OR treating OR treated OR treat OR management OR pattern OR patterns OR inpatient OR outpatient OR hospital OR medical OR insurance OR out-of-pocket OR service OR services OR drug OR drugs OR pharmaceutical OR pharmaceuticals OR resource OR resources OR therapy OR coverage OR health OR healthcare OR health-care OR diagnosis OR diagnostic OR medicine OR medicines OR medication OR prevention OR vaccination OR vaccine OR screening OR screen OR analysis OR vaccine OR utilization OR allocation OR illness OR illnesses OR evaluation OR impact OR saving OR savings OR containment OR prophylaxis OR surgery OR rehabilitation OR generic OR generics OR biosimilar OR biosimilars OR injection OR injections OR implant OR implants OR implantation OR psychiatry OR technology OR assessment ) AND ( AFFILCOUNTRY ( turkey OR jordan OR leban* OR palestin* OR gaza OR "west bank" ) ) AND ( LIMIT-TO ( SUBJAREA , "MEDI" ) OR LIMIT-TO ( SUBJAREA , "SOCI" ) OR LIMIT-TO ( SUBJAREA , "PHAR" ) OR LIMIT-TO ( SUBJAREA , "DECI" ) OR LIMIT-TO ( SUBJAREA , "NURS" ) OR LIMIT-TO ( SUBJAREA , "HEAL" ) OR LIMIT-TO ( SUBJAREA , "ECON" ) OR LIMIT-TO ( SUBJAREA , "DENT" ) ) AND ( LIMIT-TO ( PUBYEAR , 2018 ) ) | 146 |
| customTI | TITLE ( ( expenditure* OR expense* OR fund* OR budget* OR spend* OR price* OR financ* OR cost* OR economic* OR macroeconomic* OR pharmacoeconomic* OR affordab* OR purchas* OR reimburse* OR pre-pay* OR co-pay* OR copay* OR insurance OR market* OR "cost-effective*" OR "cost-utility" OR "cost-benefit" OR "economic evaluation" OR "cost per death" OR "cost per case" OR "cost per infection" OR "cost per life" OR "cost per disability-adjusted" OR "cost per quality-adjusted" OR "cost per qaly" OR qaly OR daly OR "technology assessment" OR performance OR productiv* OR efficien* OR "benefit-incidence" OR "data envelopment" OR "stochastic frontier" OR malmquist OR "Pabon-Lasso" ) AND ( health OR medical OR medicine* OR medication OR disease* OR illness OR treatment OR therap* OR pharmaceutical* OR *therap OR prevention OR prescription* OR drug* OR pharmaceutical* OR inpatient OR outpatient OR evaluation OR surgery OR surgical OR immuniz* OR immunis* OR screening OR hospital* OR physician* OR utility OR utilities OR "discrete choice" OR "contingent valuation" OR "patient-reported" OR prom ) ) AND ( AFFILCOUNTRY ( turkey OR jordan OR leban* OR palestin* OR gaza OR "west bank" ) ) AND ( LIMIT-TO ( SUBJAREA , "MEDI" ) OR LIMIT-TO ( SUBJAREA , "SOCI" ) OR LIMIT-TO ( SUBJAREA , "PHAR" ) OR LIMIT-TO ( SUBJAREA , "DECI" ) OR LIMIT-TO ( SUBJAREA , "NURS" ) OR LIMIT-TO ( SUBJAREA , "HEAL" ) OR LIMIT-TO ( SUBJAREA , "ECON" ) OR LIMIT-TO ( SUBJAREA , "DENT" ) ) AND ( LIMIT-TO ( PUBYEAR , 2018 ) ) | 157 |
| customTIABKEY_proximity7 | TITLE-ABS-KEY ( ( expenditure* OR expense* OR fund* OR budget* OR spend* OR price* OR financ* OR cost* OR economic* OR macroeconomic* OR pharmacoeconomic* OR affordab* OR purchas* OR reimburse* OR pre-pay* OR co-pay* OR copay* OR insurance OR market* OR "cost-effective*" OR "cost-utility" OR "cost-benefit" OR "economic evaluation" OR "cost per death" OR "cost per case" OR "cost per infection" OR "cost per life" OR "cost per disability-adjusted" OR "cost per quality-adjusted" OR "cost per qaly" OR qaly OR daly OR "technology assessment" OR performance OR productiv* OR efficien* OR "benefit-incidence" OR "data envelopment" OR "stochastic frontier" OR malmquist OR "Pabon-Lasso" ) W/7 ( health OR medical OR medicine* OR medication OR disease* OR illness OR treatment OR therap* OR pharmaceutical* OR *therap OR prevention OR prescription* OR drug* OR pharmaceutical* OR inpatient OR outpatient OR evaluation OR surgery OR surgical OR immuniz* OR immunis* OR screening OR hospital* OR physician* OR utility OR utilities OR "discrete choice" OR "contingent valuation" OR "patient-reported" OR prom ) ) AND ( AFFILCOUNTRY ( turkey OR jordan OR leban* OR palestin* OR gaza OR "west bank" ) ) AND ( LIMIT-TO ( SUBJAREA , "MEDI" ) OR LIMIT-TO ( SUBJAREA , "SOCI" ) OR LIMIT-TO ( SUBJAREA , "PHAR" ) OR LIMIT-TO ( SUBJAREA , "DECI" ) OR LIMIT-TO ( SUBJAREA , "NURS" ) OR LIMIT-TO ( SUBJAREA , "HEAL" ) OR LIMIT-TO ( SUBJAREA , "ECON" ) OR LIMIT-TO ( SUBJAREA , "DENT" ) ) AND ( LIMIT-TO ( PUBYEAR , 2018 ) ) | 840 |
| customTIABKEY | TITLE-ABS-KEY ( ( expenditure* OR expense* OR fund* OR budget* OR spend* OR price* OR financ* OR cost* OR economic* OR macroeconomic* OR pharmacoeconomic* OR affordab* OR purchas* OR reimburse* OR pre-pay* OR co-pay* OR copay* OR insurance OR market* OR "cost-effective*" OR "cost-utility" OR "cost-benefit" OR "economic evaluation" OR "cost per death" OR "cost per case" OR "cost per infection" OR "cost per life" OR "cost per disability-adjusted" OR "cost per quality-adjusted" OR "cost per qaly" OR qaly OR daly OR "technology assessment" OR performance OR productiv* OR efficien* OR "benefit-incidence" OR "data envelopment" OR "stochastic frontier" OR malmquist OR "Pabon-Lasso" ) AND ( health OR medical OR medicine* OR medication OR disease* OR illness OR treatment OR therap* OR pharmaceutical* OR *therap OR prevention OR prescription* OR drug* OR pharmaceutical* OR inpatient OR outpatient OR evaluation OR surgery OR surgical OR immuniz* OR immunis* OR screening OR hospital* OR physician* OR utility OR utilities OR "discrete choice" OR "contingent valuation" OR "patient-reported" OR prom ) ) AND ( AFFILCOUNTRY ( turkey OR jordan OR leban* OR palestin* OR gaza OR "west bank" ) ) AND ( LIMIT-TO ( SUBJAREA , "MEDI" ) OR LIMIT-TO ( SUBJAREA , "SOCI" ) OR LIMIT-TO ( SUBJAREA , "PHAR" ) OR LIMIT-TO ( SUBJAREA , "DECI" ) OR LIMIT-TO ( SUBJAREA , "NURS" ) OR LIMIT-TO ( SUBJAREA , "HEAL" ) OR LIMIT-TO ( SUBJAREA , "ECON" ) OR LIMIT-TO ( SUBJAREA , "DENT" ) ) AND ( LIMIT-TO ( PUBYEAR , 2018 ) ) | 2474 |

Below are further details of the findings from the bibliometric analysis.

Table A1.3 Details of the screening process, by database

| **database** | **total** | **unique_db** | **unique** | **he** | **ingeo_he** | **unique_ingeo**  **_he** | **% sens** | **% spec** |
| --- | --- | --- | --- | --- | --- | --- | --- | --- |
| Scopus | 3,822 | 3,779 | 3,769 | 325 | 316 | 316 | 56% | 8.5% |
| WOS | 3,126 | 3,113 | 1,307 | 458 | 359 | 121 | 21% | 14.7% |
| Medline | 2,072 | 2,068 | 680 | 293 | 234 | 8 | 1% | 14.1% |
| Embase | 5,470 | 5,277 | 2,317 | 632 | 577 | 113 | 20% | 11.6% |
| GlobalHealth | 625 | 624 | 273 | 74 | 73 | 8 | 1% | 11.8% |
| Econlit | 52 | 52 | 44 | 4 | 4 | 0 | 0% | 7.7% |
| NHSEED | 18 | 18 | 11 | 7 | 3 | 0 | 0% | 38.9% |
| TOTAL | **15,185** | **14,931** | **8,401** | **1,793** | **1,566** | **566** | **100%** |  |

Abbreviations: WOS – Web of Science. NHS EED – NHS Economic Evaluation Database.

Notes: Records were sorted by database in the order specified in column 1 (Scopus, WOS, Medline, Embase, GlobalHealth, Econlit, NHSEED) before de-duplication. “unique_db” denotes the number of unique records returned by searching each database as a limited number of records may appear twice; “unique” denotes number of records which were not returned by searching the databases listed above – for example, Medline returned 680 records which had not been found in Scopus and WOS; “ingeo_he” refers to articles with a central health economics topic and at least one author affiliated with an institution in either Jordan, Lebanon, OPT or Turkey; “unique_ingeo_he” is analogous to “unique” for “ingeo_he”; “%sens” and “%spec” are calculated as for Table A1.2.

Table A1.4 disaggregates the bibliometric characteristics by jurisdiction:

- Research outputs from **Jordan** (n=38) tend to be journal articles (n=36, 95%) by authors from a single organization or small international collaborations of up to three organizations (n=32, 84%), mostly with academic involvement (n=35, 92%) and low participation of other types of organizations.
- Research outputs from **Lebanon** (n=53) also tend to be journal articles, albeit to a slightly lower extent (n=32, 60%) resulting from collaborations of three or more organizations (n=32, 82% of collaborations), both academic but also public administration and industry organizations (17% and 19%, respectively).
- Research outputs from **OPT** (n=15) are all collaborations with academic involvement, overwhelmingly with international participation (n=13) and near-exclusively journal articles.
- Research outputs from **Turkey** (n=464) tend to be either single organization studies or small international collaborations of up to three organizations (n=319, 69%), predominantly by authors from Turkish organizations (70%) and with a significant share of conference presentations (n=92, 20%).

Absolute numbers (# records) can be misleading because the four jurisdictions are very different in terms of economic and population size. Table A1.5 presents adjusted figures as number of co-authored records per 1,000 USD in GDP per capita and as number of co-authored records per 10,000 population. Turkish organizations remain the most productive and organizations in OPT the least productive under both adjustments. Lebanon and Jordan are the second most productive depending on adjustment (Jordan if adjusting for GDP per capita, Lebanon if adjusting for population size).

The total number of records with authorship from an organization in a given jurisdiction were presented both unadjusted and adjusted for GDP per capita and population size for 2016 (World Bank Group 2019), the mid-year in this study’s time scope. Ideally, volumes would be adjusted by number of researchers per million population using UNESCO data (UNESCO Institute of Statistics 2019), but this was not possible as only Turkey had available data, hence we adjusted using GDP per capita and population size following Cash-Gibson et al (Cash-Gibson, et al. 2018).

Figure A1.2 below illustrates the co-publication patterns across organizations. There is a rich and diverse network of more than 30 Turkish organizations (left of the graph) – the network is centred around several Turkish universities which contribute the largest number of records (e.g. Hacettepe U, Ankara U, Gazi U) which co-publish with other universities (both Turkish and foreign, e.g. Columbia University), pharmaceutical companies (Turkish and international offices), local consultancies and teaching hospitals. There are much fewer Lebanese and Jordanian organizations (green cluster to the right of the graph) – with AUB and Jordan University of Science and Technology more prominent.

Table A1.4 Bibliometric characteristics of included records, by jurisdiction (n=566)

|  | Jordan (n=38) | | Lebanon (n=53) | | OPT (n=15) | | Turkey (n=464) | |
| --- | --- | --- | --- | --- | --- | --- | --- | --- |
|  | N | % | N | % | N | % | N | % |
| # organizations per record |  |  |  |  |  |  |  |  |
| One | 14 | 36.8% | 14 | 26.4% | 0 | 0.0% | 132 | 28.4% |
| Two | 10 | 26.3% | 7 | 13.2% | 5 | 33.3% | 111 | 23.9% |
| Three | 8 | 21.1% | 10 | 18.9% | 2 | 13.3% | 76 | 16.4% |
| Four | 3 | 7.9% | 8 | 15.1% | 2 | 13.3% | 43 | 9.3% |
| Five+ | 3 | 7.9% | 14 | 26.4% | 6 | 40.0% | 102 | 22.0% |
|  |  |  |  |  |  |  |  |  |
| Organization type per record |  |  |  |  |  |  |  |  |
| Academic | 35 | 92.1% | 51 | 96.2% | 15 | 100.0% | 409 | 88.1% |
| Clinical | 7 | 18.4% | 12 | 22.6% | 5 | 33.3% | 141 | 30.4% |
| Public institution | 3 | 7.9% | 9 | 17.0% | 3 | 20.0% | 41 | 8.8% |
| Industry (pharm/consulting) | 2 | 5.3% | 10 | 18.9% | 1 | 6.7% | 117 | 25.2% |
| International organization | 3 | 7.9% | 2 | 3.8% | 2 | 13.3% | 3 | 0.6% |
|  |  |  |  |  |  |  |  |  |
| Of records with 2+ organizations | 24 |  | 39 |  | 15 |  | 332 |  |
| International collaborations | 20 | 83.3% | 32 | 82.1% | 13 | 86.7% | 140 | 42.2% |
| Domestic institutions only | 4 | 16.7% | 7 | 17.9% | 2 | 13.3% | 192 | 57.8% |
|  |  |  |  |  |  |  |  |  |
| Type of record |  |  |  |  |  |  |  |  |
| Journal article | 36 | 94.7% | 46 | 86.8% | 14 | 93.3% | 370 | 79.7% |
| Conference proceeding | 1 | 2.6% | 7 | 13.2% | 1 | 6.7% | 92 | 19.8% |
| Book or book chapter | 1 | 2.6% | 0 | 0.0% | 0 | 0.0% | 2 | 0.4% |
|  |  |  |  |  |  |  |  |  |
| Top 5 journals (# articles) | jordan journal of pharmaceutical sciences (4), value in health (4), intl journal of health planning and management (2), journal of laparoendoscopic and advanced surgical techniques (1), bmc health services research (1) | | plos one (3), the lancet (3), current therapeutic research - clinical and experimental (2), journal medical libanais (2), value in health regional issues (2) | | the lancet (2), journal of occupational and environmental medicine (2), inquiry (1), value in health (1), conflict and health (1) | | value in health (65), tuberkuloz ve toraks (5), turkish journal of medical sciences (5), intl journal of health planning and management (4), taf preventive medicine bulletin (4) | |

Table A1.5 Adjusted productivity ratios, by jurisdiction

|  | # records | GDP per capita 2016 (PPP constant 2011 dollars) | Records per 1,000 USD per capita | Total population 2016 | Records per 10,000 population |
| --- | --- | --- | --- | --- | --- |
| Jordan | 38 | 8,390 | 4.53 | 9,455,802 | 8.87 |
| Lebanon | 53 | 13,156 | 2.89 | 6,006,668 | 13.97 |
| OPT | 15 | 23,756 | 1.60 | 79,512,426 | 1.06 |
| Turkey | 464 | 4,738 | 8.02 | 4,551,566 | 18.43 |

Sources: GDP per capita and Total population from <https://data.worldbank.org/>

Figure A1.1 Cross-jurisdiction co-publication patterns, all included records (n=566)


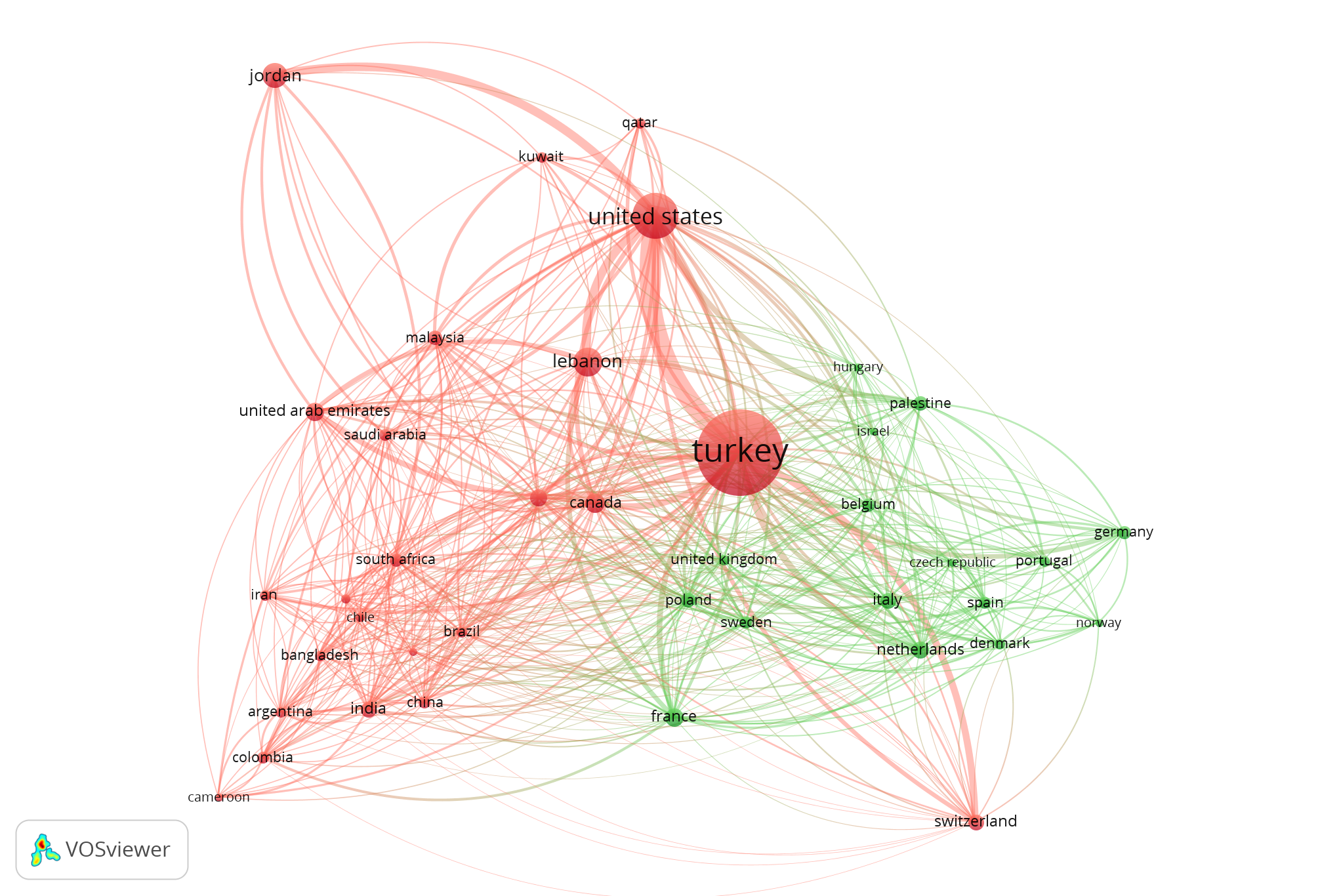


Notes: only countries appearing in at least 5 records are displayed; minimum 10 countries per cluster

Figure A1.2 Cross-institutional co-publication patterns, all included records (n=566)


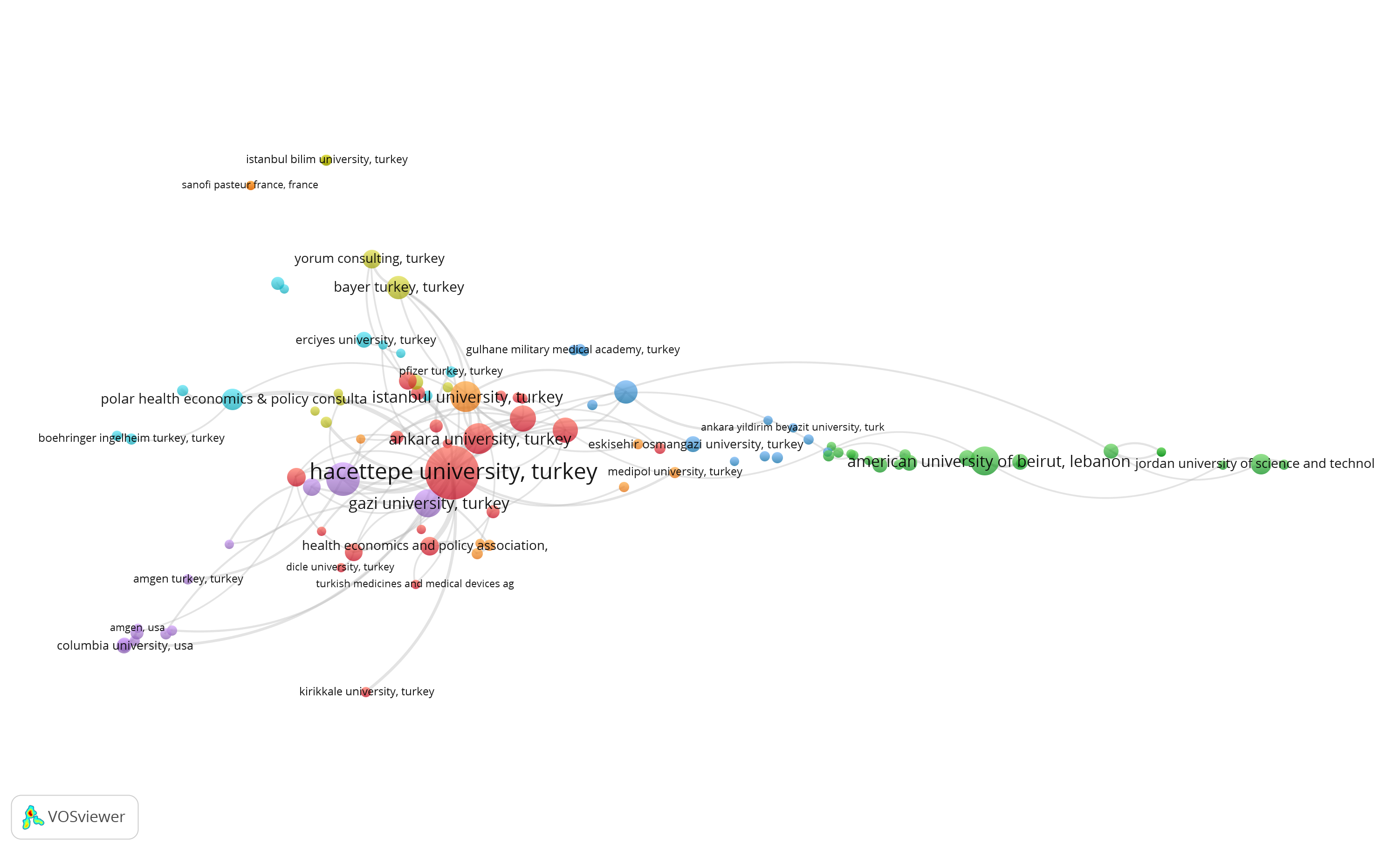


Notes: only organizations with at least 3 records are displayed; minimum 10 organizations per cluster

**Figure A1.3. Key topics of included records, excluding conference proceedings (n=464)**


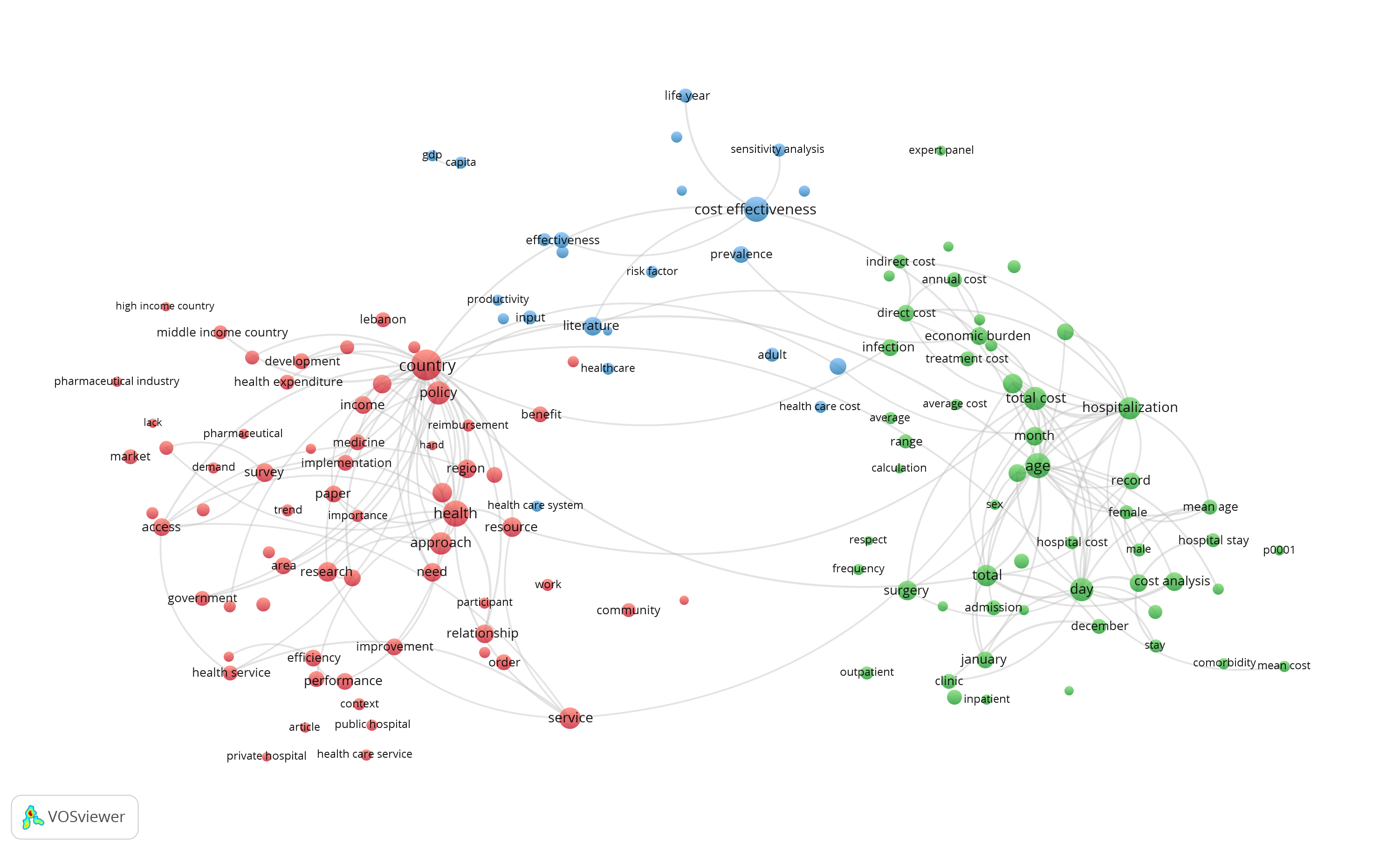


Notes: only terms with at least 10 occurrences and links with at least 10 co-occurrences are displayed; minimum 10 items per cluster.

**Figure A1.4. Key topics of included records, only conference proceedings (n=102)**


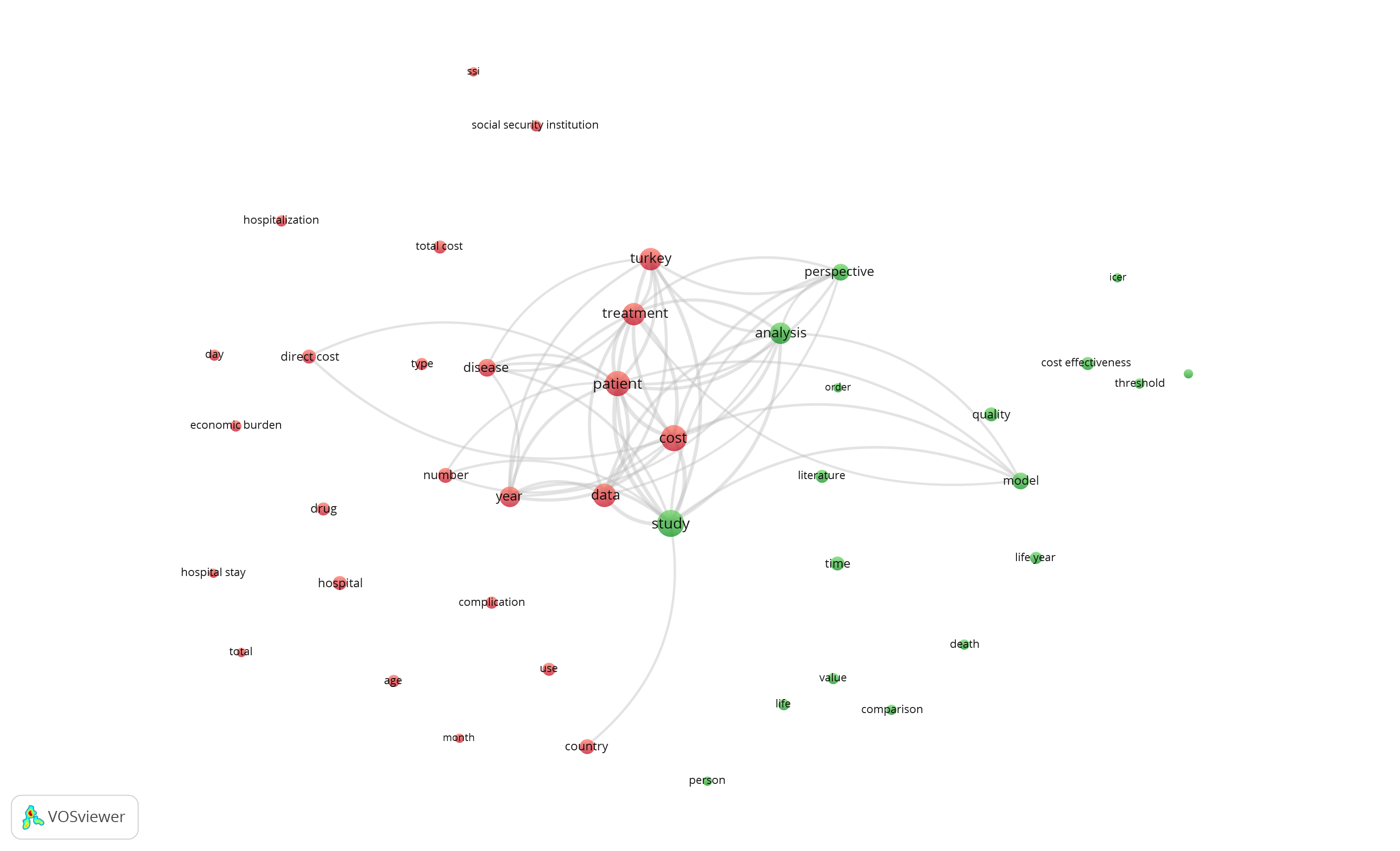


Notes: only terms with at least 10 occurrences and links with at least 20 co-occurrences are displayed; minimum 10 items per cluster.

**Figure A1.5. Turkey – topics**


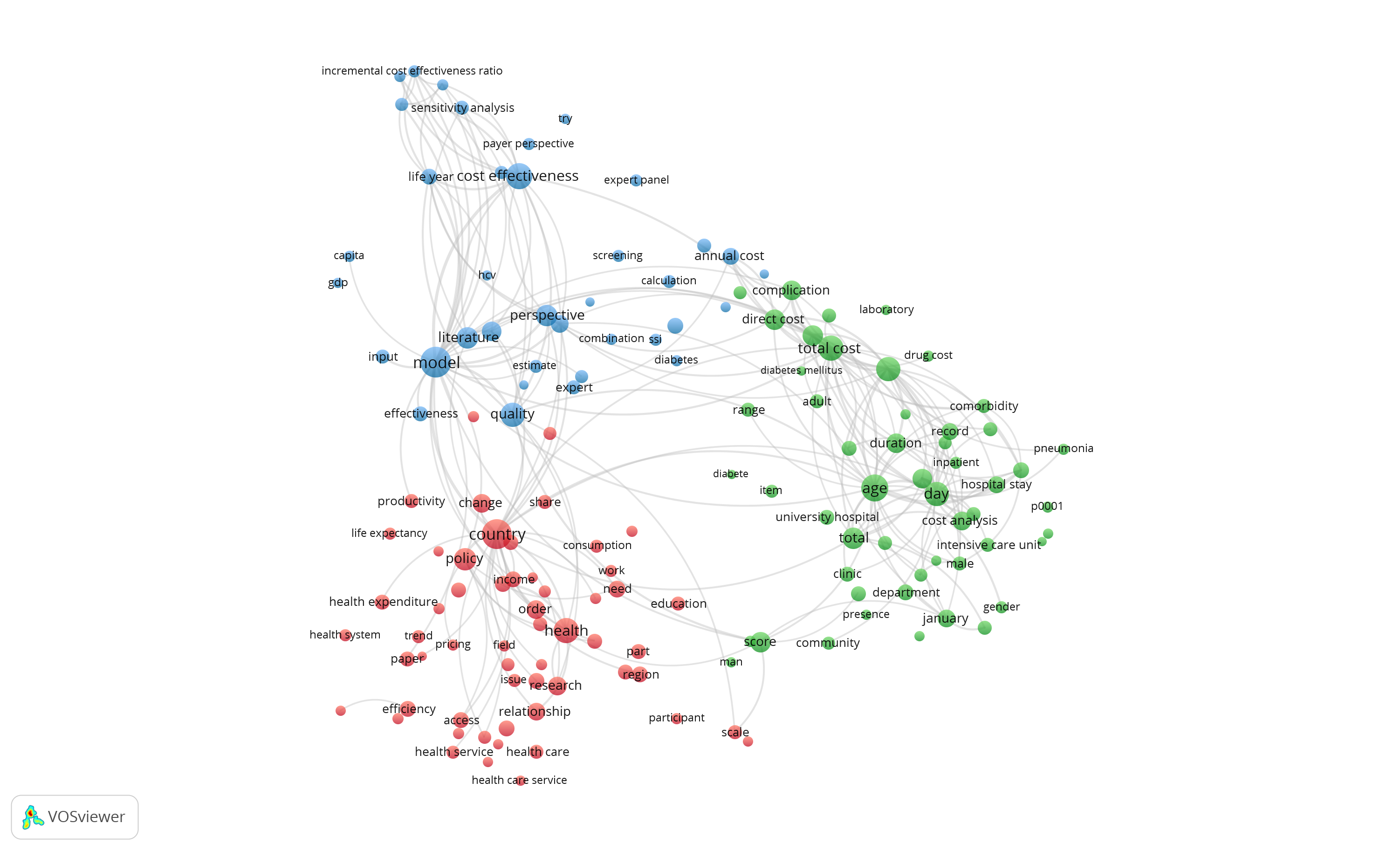


Notes: only terms with at least 10 occurrences and links with at least 10 co-occurrences are displayed; minimum 10 items per cluster.

**Figure A1.6. Turkey – organizations**


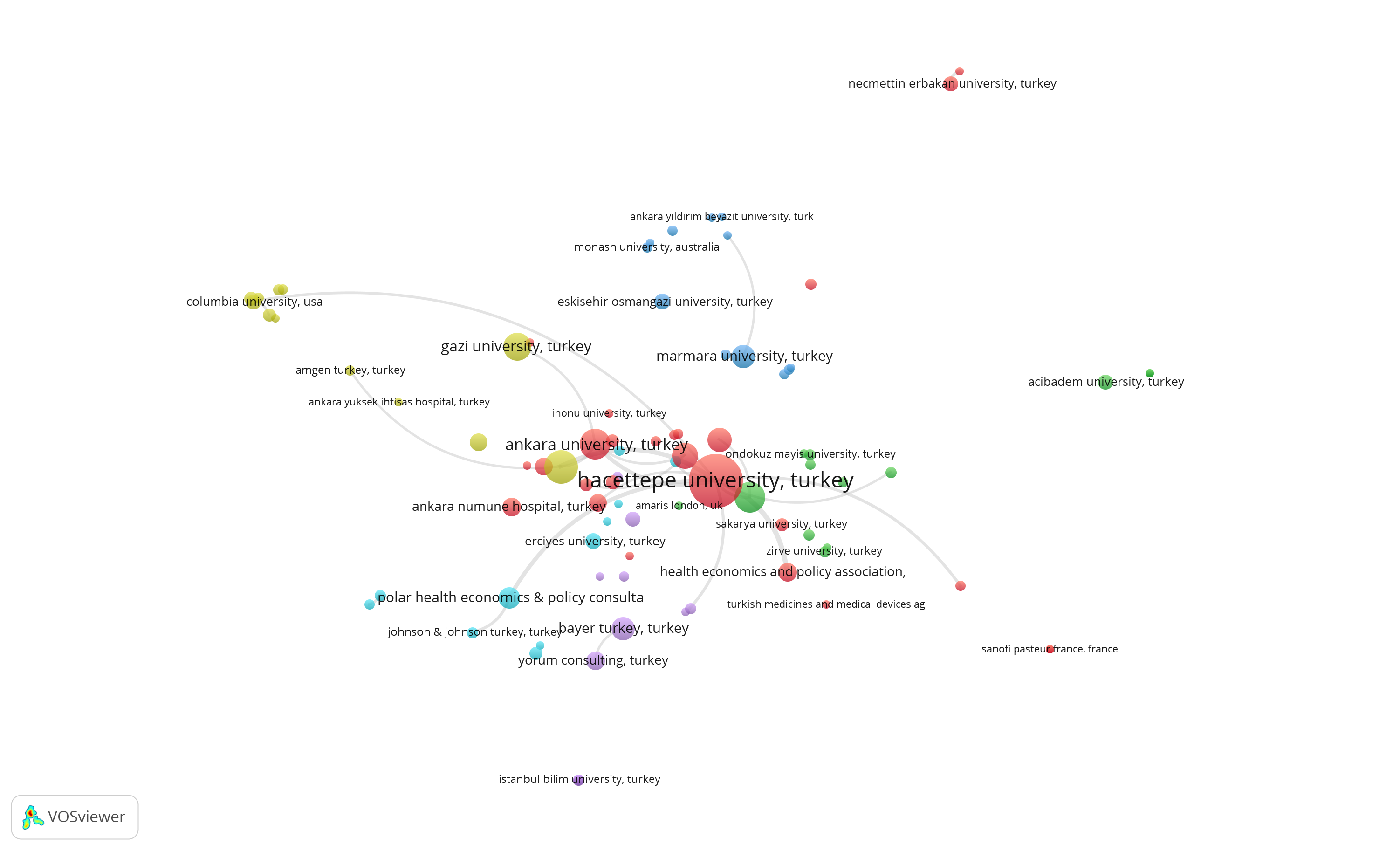


Notes: only organizations with at least 3 occurrences are displayed; minimum 10 organizations per cluster.

**Figure A1.7. Turkey – countries**


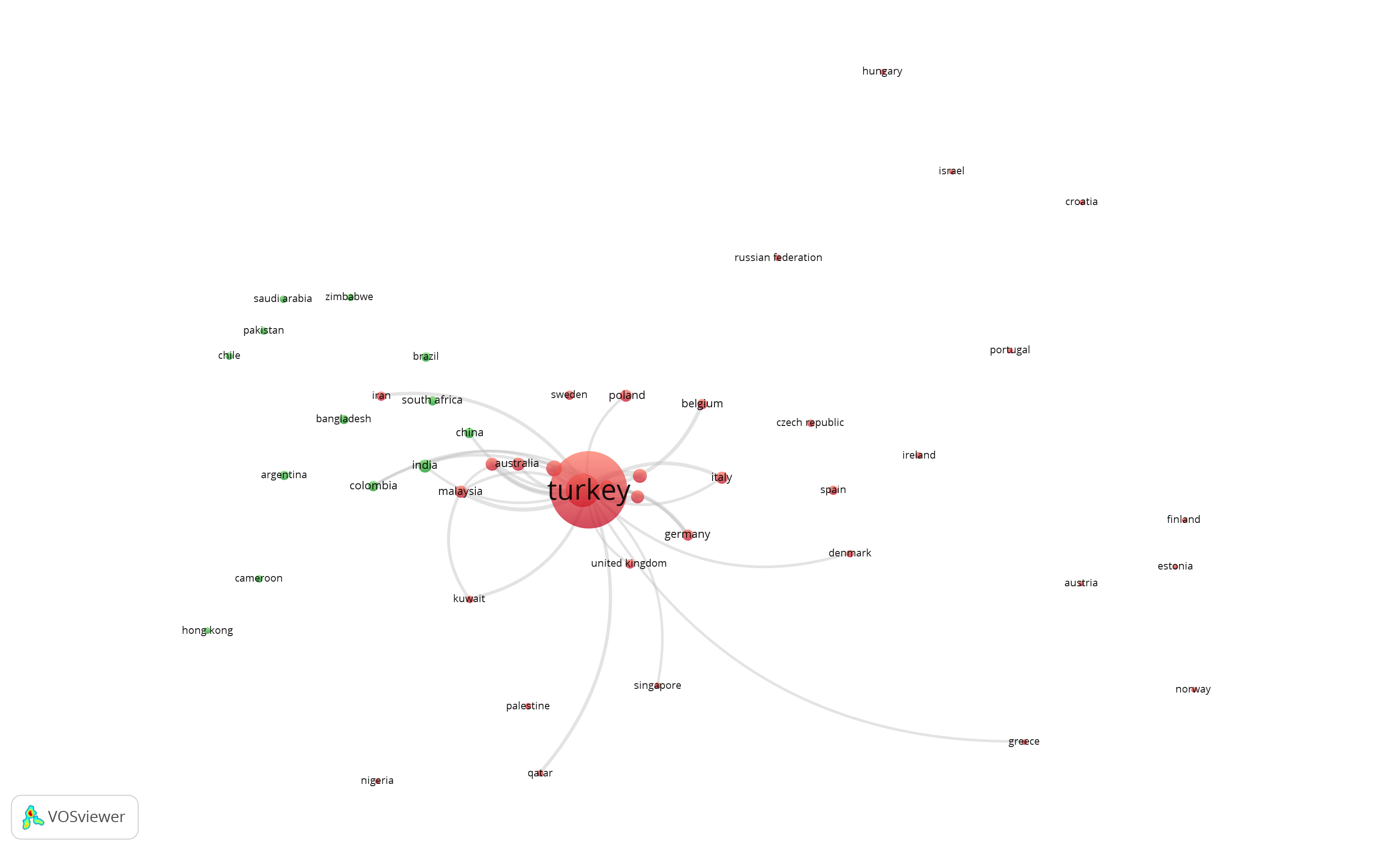


Notes: only countries with at least 3 occurrences and links with at least 2 countries are displayed; minimum 10 countries per cluster.

**Figure A1.8. Jordan – topics**


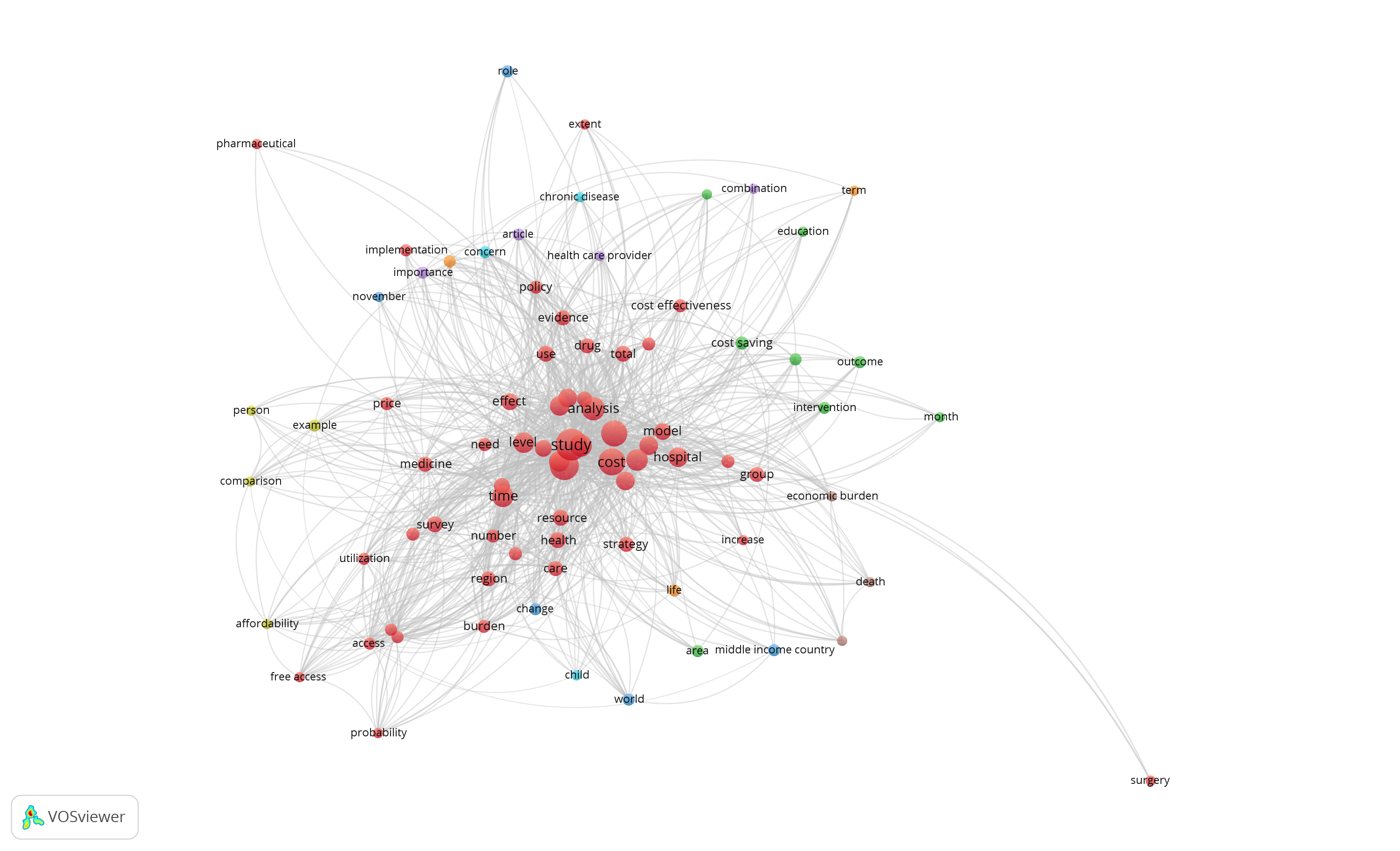


Notes: only terms with at least 3 occurrences and links with at least 2 co-occurrences are displayed; minimum 10 items per cluster.

**Figure A1.9. Jordan – organizations**


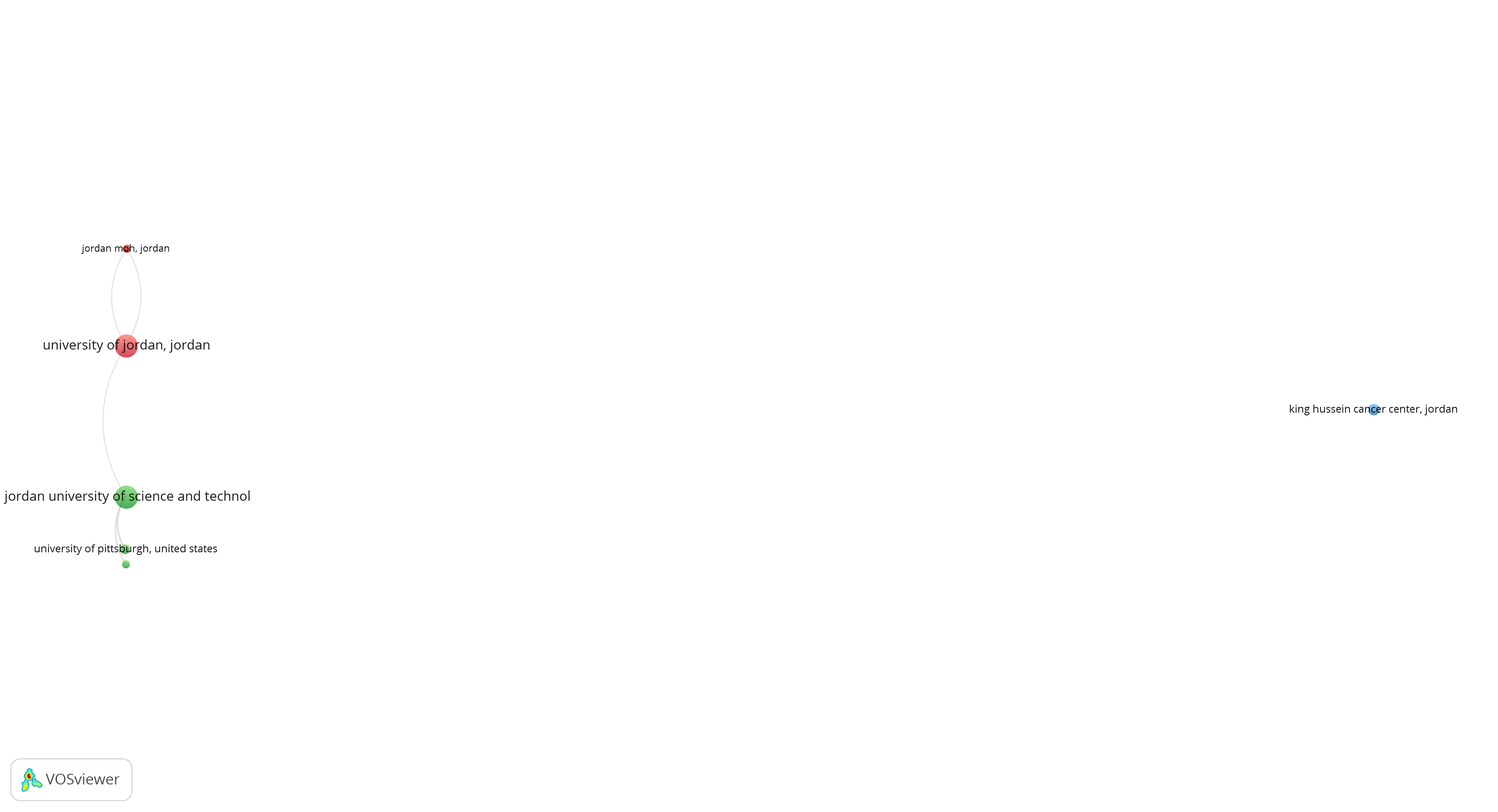


Notes: only organizations with at least 2 occurrences are displayed; minimum 3 organizations per cluster.

**Figure A1.10. Jordan – countries**


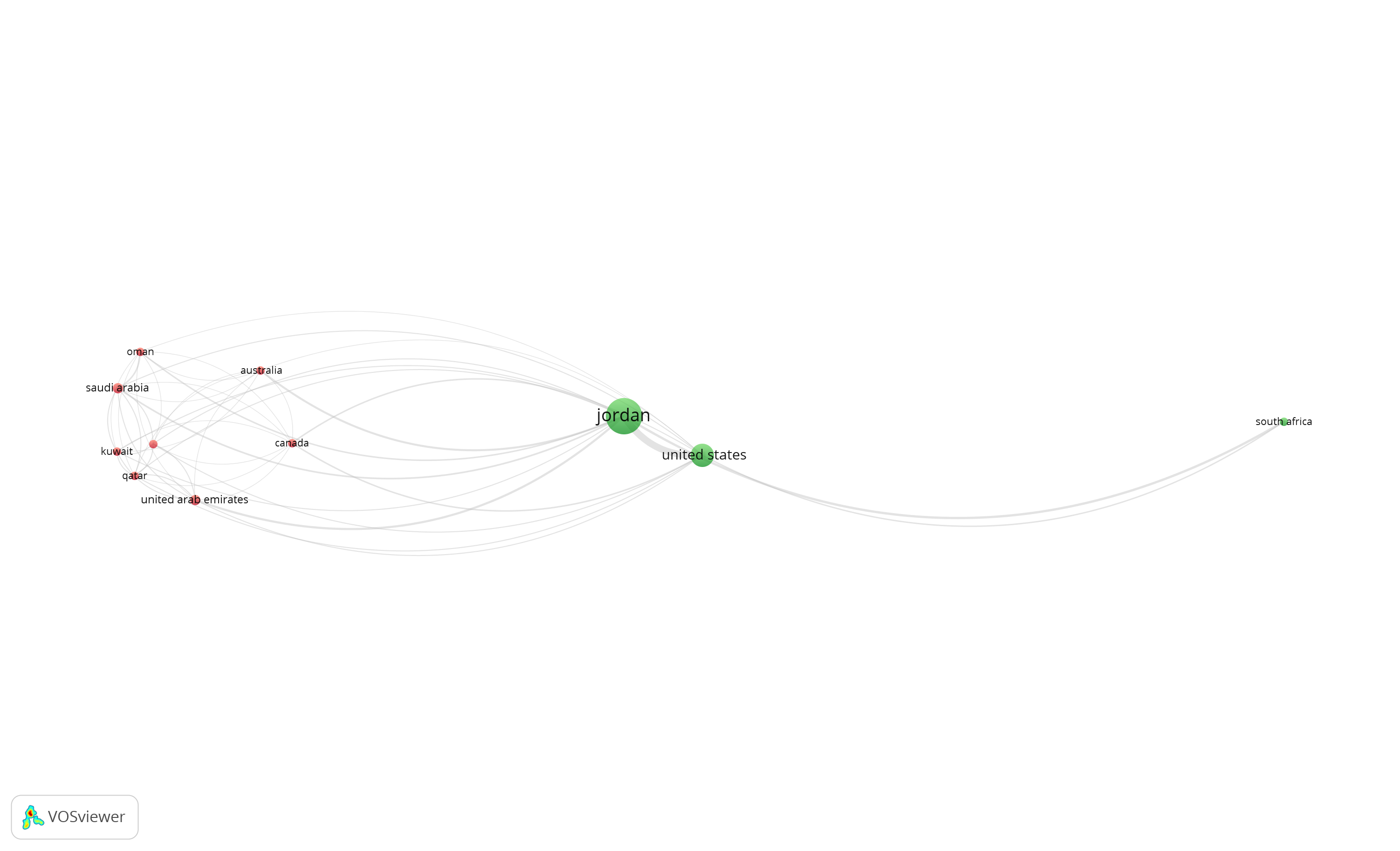


Notes: only countries with at least 3 occurrences are displayed; minimum 3 countries per cluster.

**Figure A1.11. Lebanon – topics**


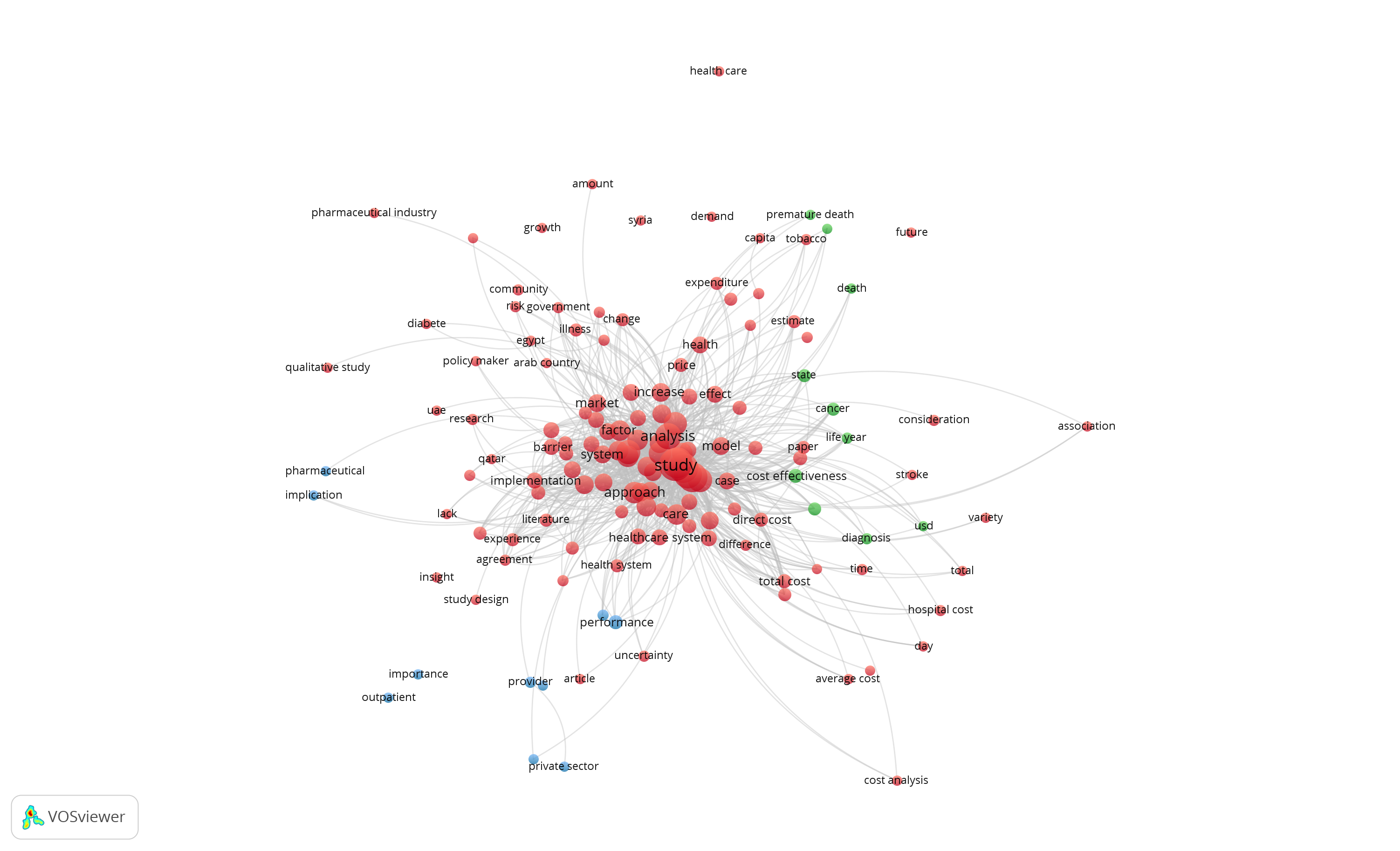


Notes: only terms with at least 3 occurrences and links with at least 2 co-occurrences are displayed; minimum 10 items per cluster.

**Figure A1.12. Lebanon – organizations**


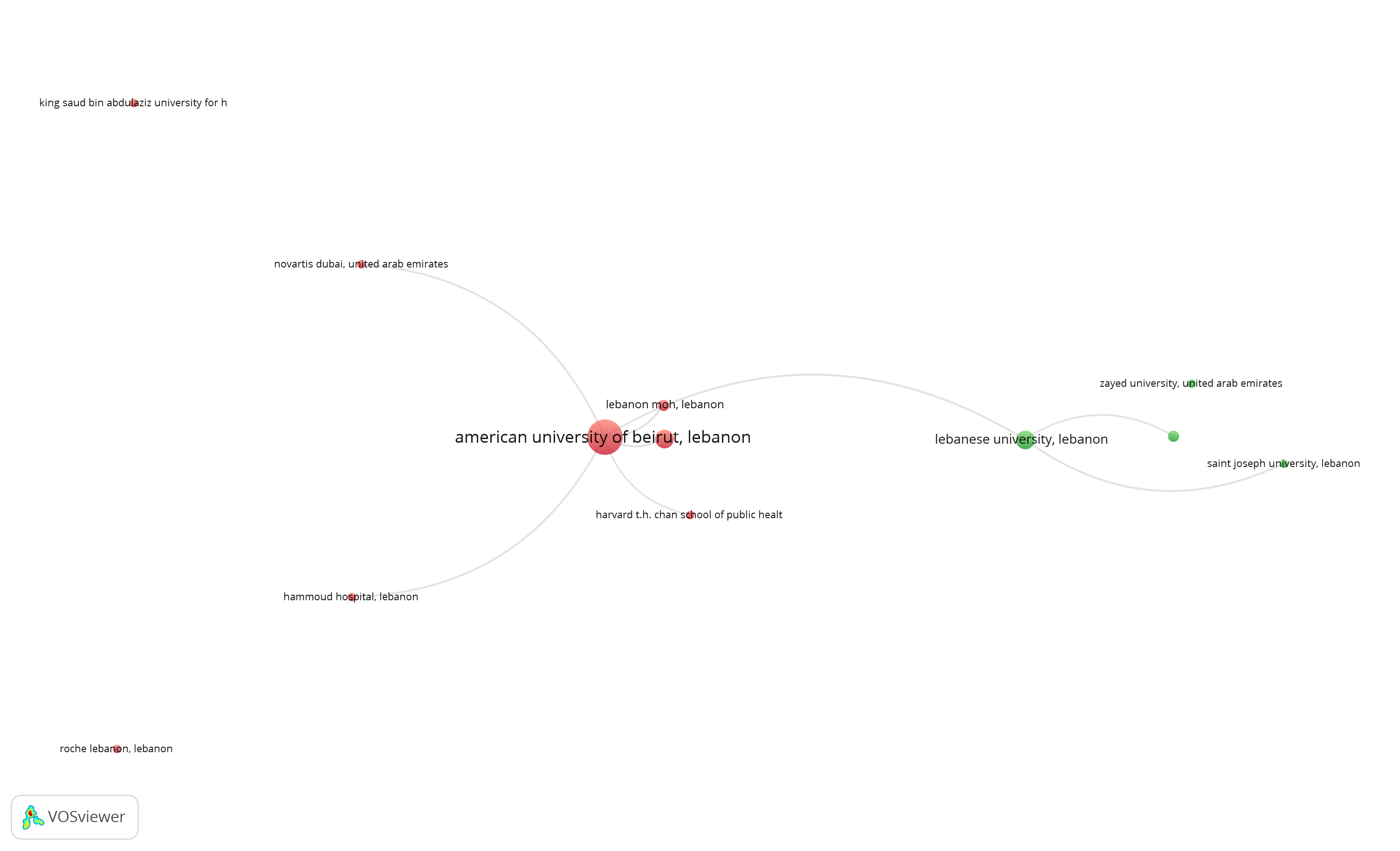


Notes: only organizations with at least 2 occurrences are displayed; minimum 3 organizations per cluster.

**Figure A1.13. Lebanon – countries**


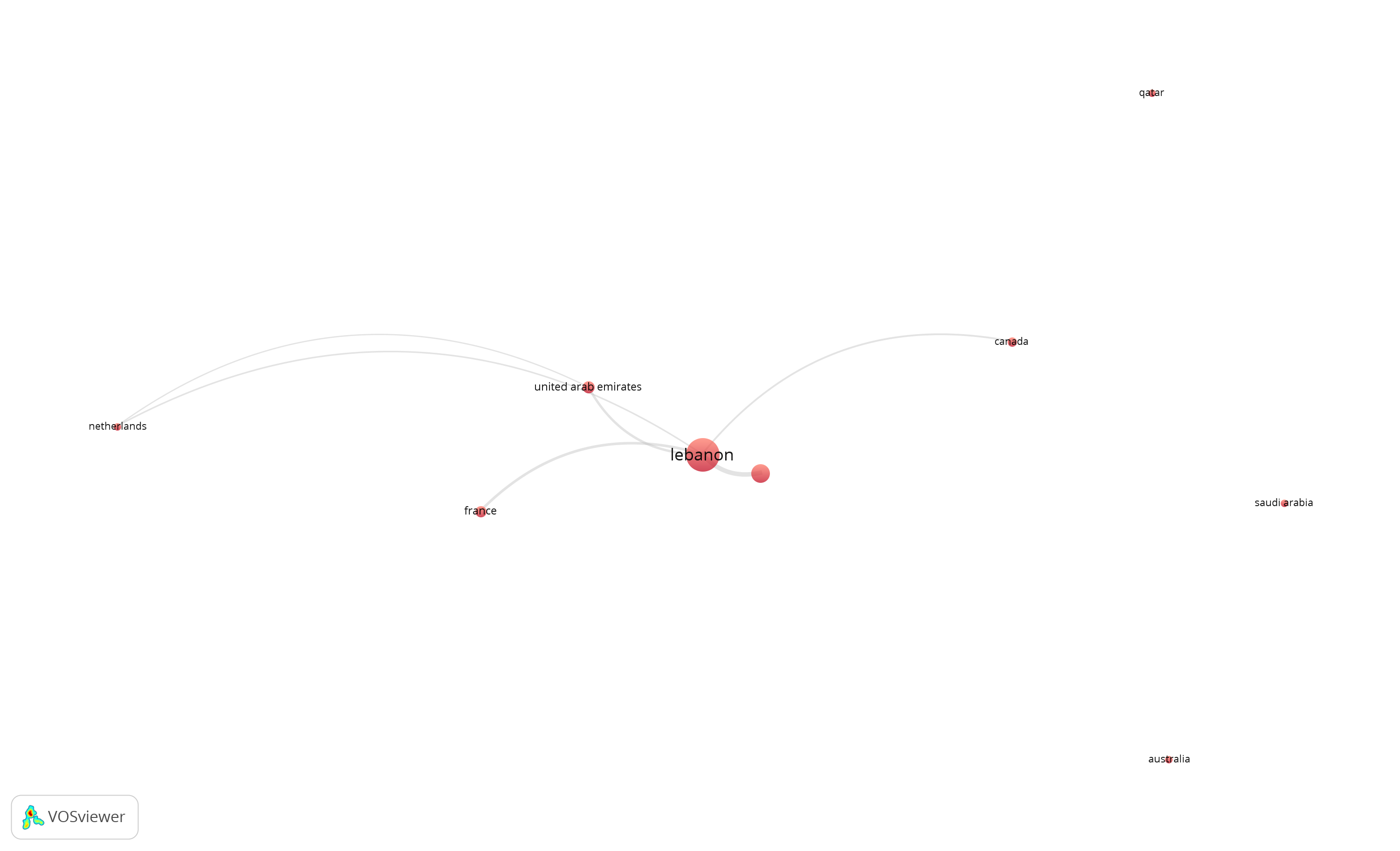


Notes: only countries with at least 3 occurrences are displayed; minimum 3 countries per cluster.

**Figure A1.14. OPT – topics**


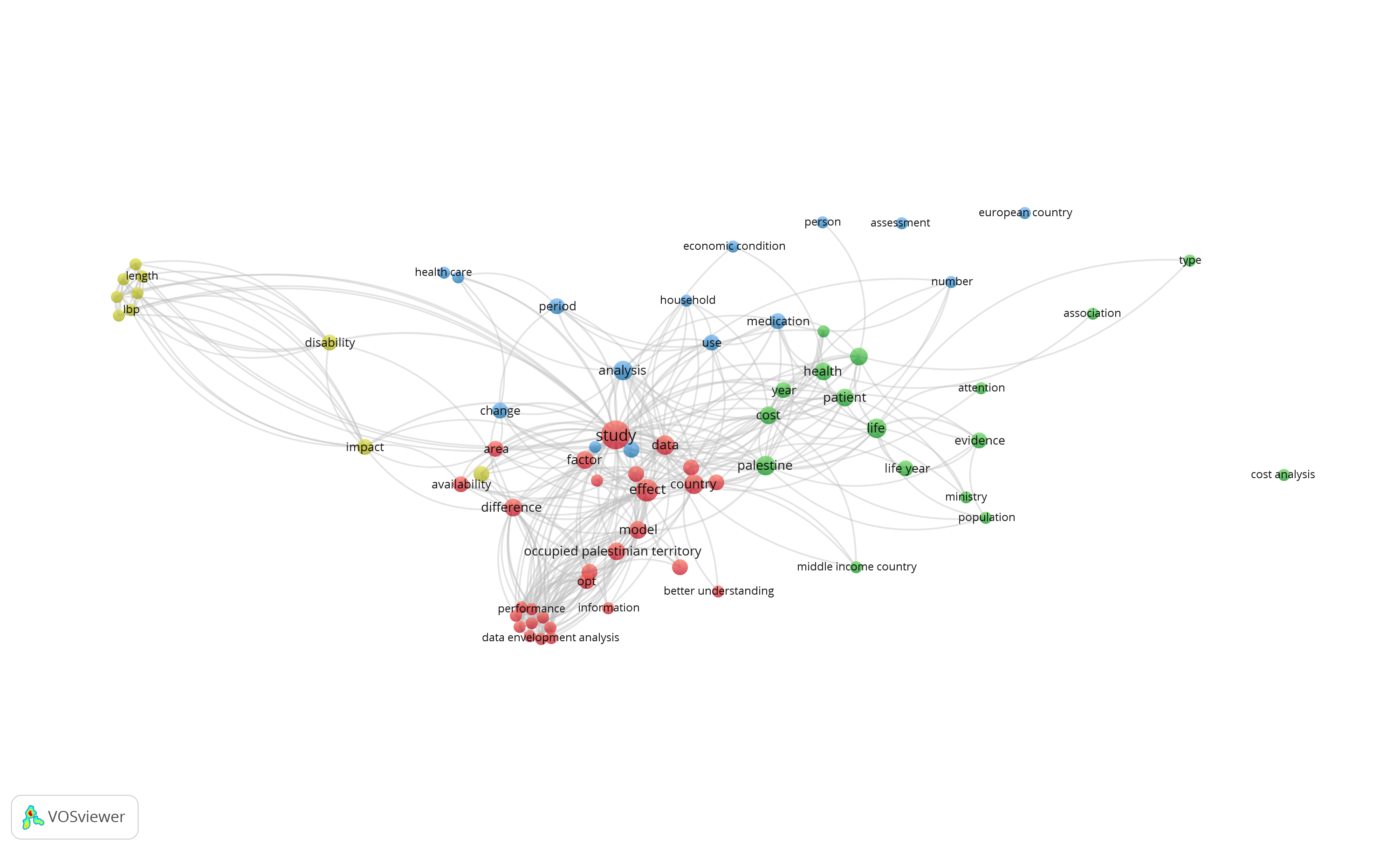


Notes: only terms with at least 3 occurrences and links with at least 2 co-occurrences are displayed; minimum 10 items per cluster.

**Figure A1.15. OPT – organizations**


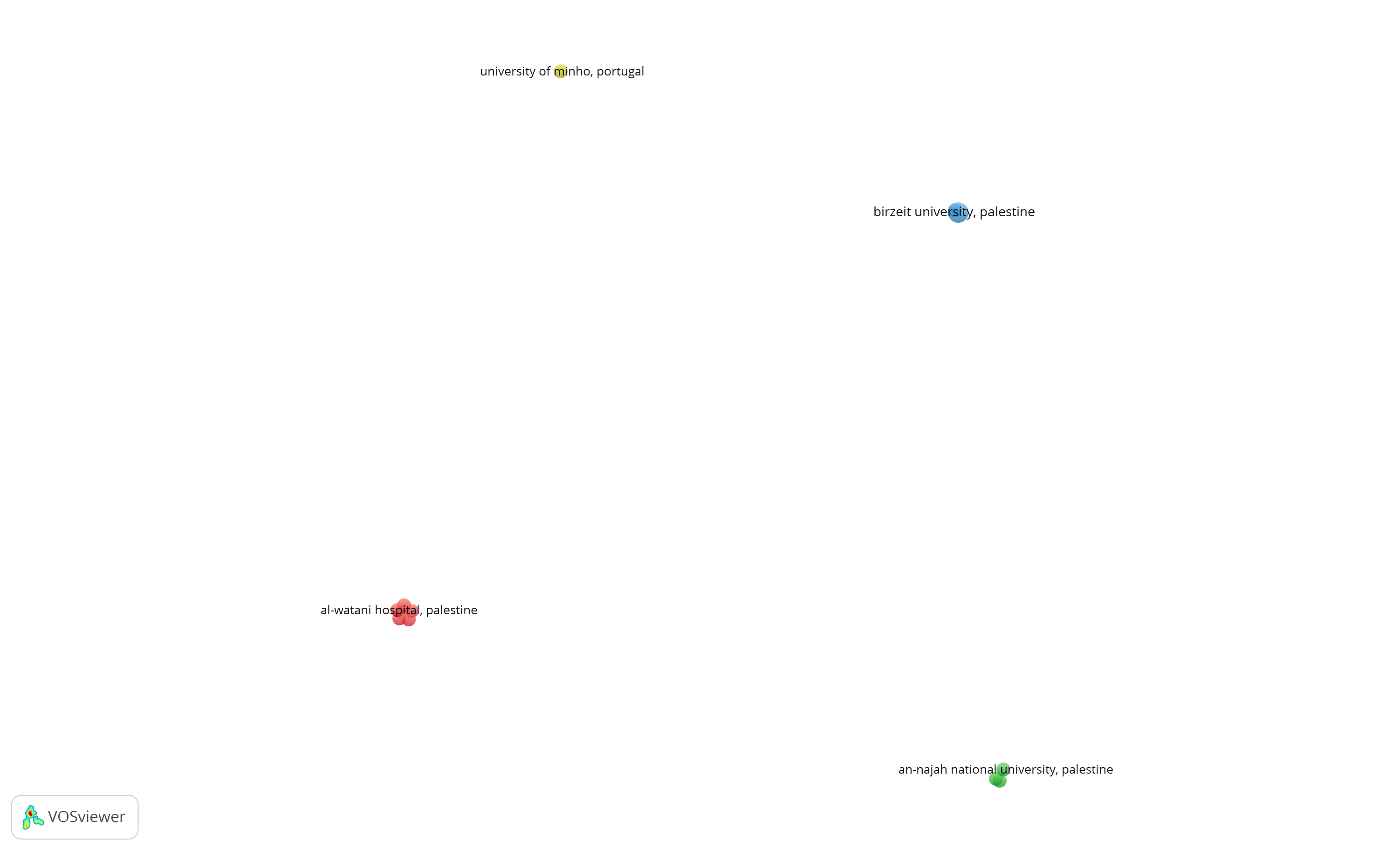


Notes: only organizations with at least 2 occurrences are displayed; minimum 3 organizations per cluster.

**Figure A1.16. OPT – countries**


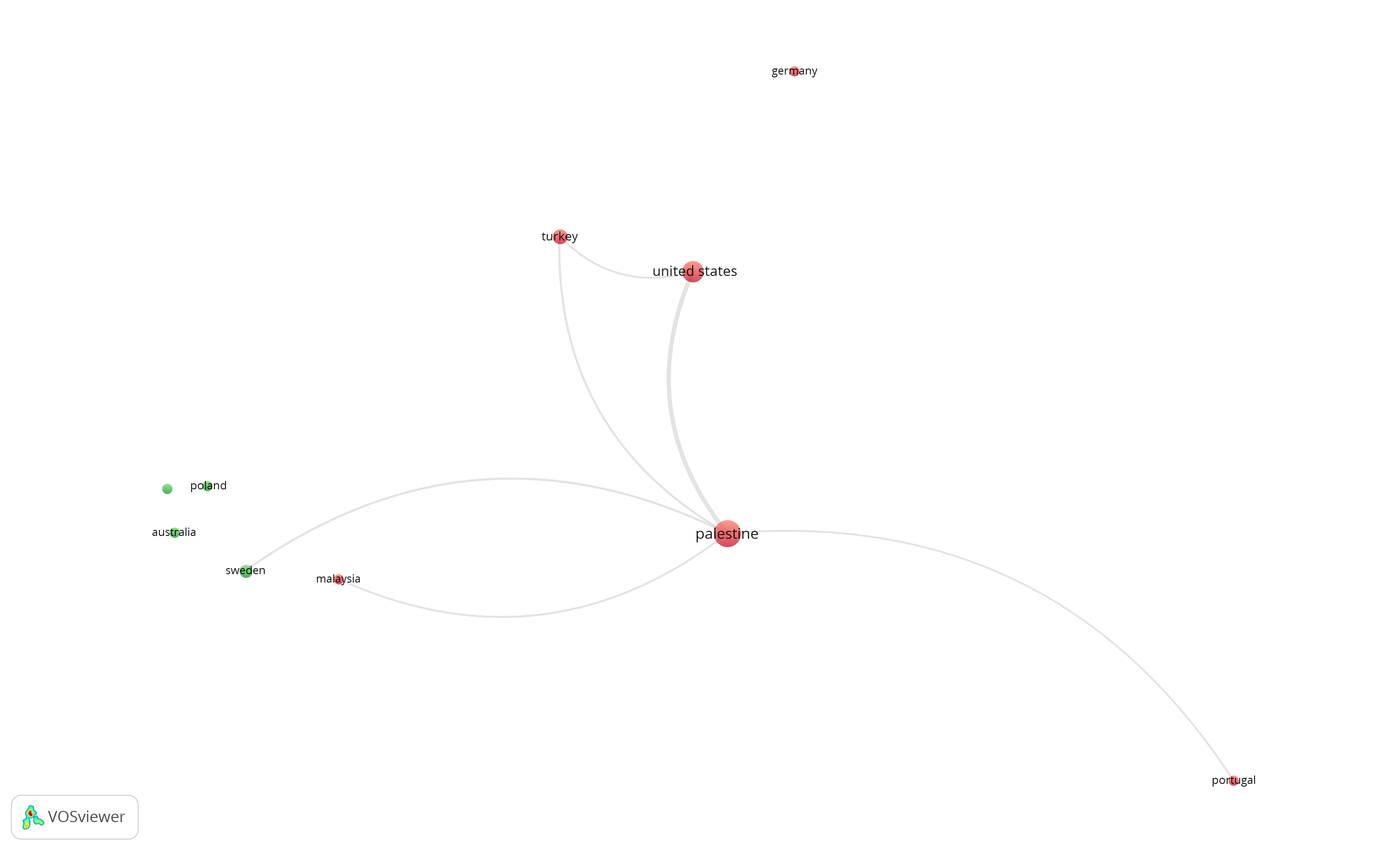


Notes: only countries with at least 3 occurrences are displayed; minimum 3 countries per cluster

# Bibliography

Cash-Gibson, L, DF Rojas-Gualdron, JM Pericas, and J Benach. 2018. "Inequalities in global health inequalities research: A 50-year bibliometric analysis (1966-2015)." *PLoS One* 13 (1): e0191901.

Jakovljevic, M, and AV Pejcic. 2017. "Growth of Global Publishing Output of Health Economics in the Twenty-First Century: A Bibliographic Insight." *Frontiers in Public Health* 5 (211).

Pitt, C, C Goodman, and K Hanson. 2016. "Economic Evaluation in Global Perspective: A Bibliometric Analysis of the Recent Literature." *Health Economics* 25 (S1): 9-28.

UNESCO Institute of Statistics. 2019. *How much does your country invest in R&D?* Accessed February 19, 2019. http://uis.unesco.org/apps/visualisations/research-and-development-spending/.

World Bank Group. 2019. *World Bank Data Catalog.* Accessed February 19, 2019. https://data.worldbank.org/.

Appendix 2. Literature search strategies used in the bibliometric analysis

All databases were searched on 28 January 2019.

Databases searched via OvidSP

1. OVID Medline(R) and In-Process & Other Non-Indexed Citations and Daily
2. Embase Classic + Embase
3. Global Health

| # | Search | Medline | Embase | Global Health |
| --- | --- | --- | --- | --- |
| 1 | ((expenditure* or expense* or fund* or budget* or spend* or price* or financ* or cost* or economic* or macroeconomic* or pharmacoeconomic* or affordab* or purchas* or reimburse* or pre-pay* or co-pay* or copay* or insurance or market* or "cost-effective*" or "cost-utility" or "cost-benefit" or "economic evaluation" or "cost per death" or "cost per case" or "cost per infection" or "cost per life" or "cost per disability-adjusted" or "cost per quality-adjusted" or "cost per qaly" or qaly or daly or "technology assessment" or performance or productiv* or efficien* or "benefit-incidence" or "data envelopment" or "stochastic frontier" or malmquist or "Pabon-Lasso") adj7 (health or medical or medicine* or medication or disease* or illness or treatment or therap* or pharmaceutical* or chemotherap* or prevention or prescription* or drug* or pharmaceutical* or inpatient or outpatient or evaluation or surgery or surgical or immuniz* or immunis* or vaccin* or screening or hospital* or physician* or utility or utilities or "discrete choice" or "contingent valuation" or "patient-reported" or prom)).mp. | 766110 | 1320512 | 142454 |
| 2 | (turkey or jordan or leban* or palestin* or gaza or "West Bank").in. | 249705 | 439133 | 50689 |
| 3 | #1 and #2 | 6127 | 14037 | 1536 |
| 4 | limit 5 to (humans and yr="2014 -Current") | 2072 | 5470 | 625 |

Note: ‘humans’ filter only applicable in Medline and Embase.

1. Econlit

| # | Search | Econlit |
| --- | --- | --- |
| 1 | ((expenditure* or expense* or fund* or budget* or spend* or price* or financ* or cost* or economic* or macroeconomic* or pharmacoeconomic* or affordab* or purchas* or reimburse* or pre-pay* or co-pay* or copay* or insurance or market* or "cost-effective*" or "cost-utility" or "cost-benefit" or "economic evaluation" or "cost per death" or "cost per case" or "cost per infection" or "cost per life" or "cost per disability-adjusted" or "cost per quality-adjusted" or "cost per qaly" or qaly or daly or "technology assessment" or performance or productiv* or efficien* or "benefit-incidence" or "data envelopment" or "stochastic frontier" or malmquist or "Pabon-Lasso") and (health or medical or medicine* or medication or disease* or illness or treatment or therap* or pharmaceutical* or chemotherap* or prevention or prescription* or drug* or pharmaceutical* or inpatient or outpatient or evaluation or surgery or surgical or immuniz* or immunis* or vaccin* or screening or hospital* or physician* or utility or utilities or "discrete choice" or "contingent valuation" or "patient-reported" or prom)).mp. | 181440 |
| 2 | (turkey or jordan or leban* or palestin* or gaza or "West Bank").in. | 1275 |
| 3 | #1 and #2 | 108 |
| 4 | limit 5 to (humans and yr="2014 -Current") | 52 |

1. Web of Science Core Collections

| # | Search | Hits |
| --- | --- | --- |
| 1 | TS=(( expenditure* OR expense* OR fund* OR budget* OR spend* OR price* OR financ* OR cost* OR economic* OR macroeconomic* OR pharmacoeconomic* OR affordab* OR purchas* OR reimburse* OR pre-pay* OR co-pay* OR copay* OR insurance OR market* OR "cost-effective*" OR "cost-utility" OR "cost-benefit" OR "economic evaluation" OR "cost per death" OR "cost per case" OR "cost per infection" OR "cost per life" OR "cost per disability-adjusted" OR "cost per quality-adjusted" OR "cost per qaly" OR qaly OR daly OR "technology assessment" OR performance OR productiv* OR efficien* OR "benefit-incidence" OR "data envelopment" OR "stochastic frontier" OR malmquist OR "Pabon-Lasso" ) NEAR/7 ( health OR medical OR medicine* OR medication OR disease* OR illness OR treatment OR therap* OR pharmaceutical* OR *therap OR prevention OR prescription* OR drug* OR pharmaceutical* OR inpatient OR outpatient OR evaluation OR surgery OR surgical OR immuniz* OR immunis* OR vaccin* OR screening OR hospital* OR physician* OR utility OR utilities OR "discrete choice" OR "contingent valuation" OR "patient-reported" OR prom ))  Indexes=SCI-EXPANDED, SSCI, A&HCI, CPCI-S, CPCI-SSH, ESCI Timespan=2014-2019 | 318004 |
| 2 | AD=(turkey OR jordan OR leban* OR palestin* OR gaza OR "west bank")  Indexes=SCI-EXPANDED, SSCI, A&HCI, CPCI-S, CPCI-SSH, ESCI Timespan=2014-2019 | 271652 |
| 3 | #2 and #1 | 6317 |
| 4 | #2 AND #1  Refined by: WEB OF SCIENCE CATEGORIES: ( GERIATRICS GERONTOLOGY OR MEDICINE GENERAL INTERNAL OR MANAGEMENT OR INFECTIOUS DISEASES OR RHEUMATOLOGY OR HEALTH CARE SCIENCES SERVICES OR OPERATIONS RESEARCH MANAGEMENT SCIENCE OR PHARMACOLOGY PHARMACY OR MICROBIOLOGY OR SURGERY OR HEALTH POLICY SERVICES OR PUBLIC ENVIRONMENTAL OCCUPATIONAL HEALTH OR CARDIAC CARDIOVASCULAR SYSTEMS OR ECONOMICS OR OBSTETRICS GYNECOLOGY OR RESPIRATORY SYSTEM OR ONCOLOGY OR OPHTHALMOLOGY OR DERMATOLOGY OR REHABILITATION OR NURSING OR OTORHINOLARYNGOLOGY OR CLINICAL NEUROLOGY OR IMMUNOLOGY OR NEUROSCIENCES OR SOCIAL SCIENCES INTERDISCIPLINARY OR MEDICAL INFORMATICS OR ALLERGY OR EMERGENCY MEDICINE OR MULTIDISCIPLINARY SCIENCES OR PSYCHIATRY OR HEMATOLOGY OR PEDIATRICS OR PERIPHERAL VASCULAR DISEASE OR UROLOGY NEPHROLOGY OR ORTHOPEDICS OR CRITICAL CARE MEDICINE OR BUSINESS OR GERONTOLOGY OR GASTROENTEROLOGY HEPATOLOGY )  Indexes=SCI-EXPANDED, SSCI, A&HCI, CPCI-S, CPCI-SSH, ESCI Timespan=2014-2019 | 3126 |

1. Scopus

| # | Search | Hits |
| --- | --- | --- |
| 1 | TITLE-ABS-KEY ( ( expenditure* OR expense* OR fund* OR budget* OR spend* OR price* OR financ* OR cost* OR economic* OR macroeconomic* OR pharmacoeconomic* OR affordab* OR purchas* OR reimburse* OR pre-pay* OR co-pay* OR copay* OR insurance OR market* OR "cost-effective*" OR "cost-utility" OR "cost-benefit" OR "economic evaluation" OR "cost per death" OR "cost per case" OR "cost per infection" OR "cost per life" OR "cost per disability-adjusted" OR "cost per quality-adjusted" OR "cost per qaly" OR qaly OR daly OR "technology assessment" OR performance OR productiv* OR efficien* OR "benefit-incidence" OR "data envelopment" OR "stochastic frontier" OR malmquist OR "Pabon-Lasso" ) W/7 ( health OR medical OR medicine* OR medication OR disease* OR illness OR treatment OR therap* OR pharmaceutical* OR *therap OR prevention OR prescription* OR drug* OR pharmaceutical* OR inpatient OR outpatient OR evaluation OR surgery OR surgical OR immuniz* OR immunis* OR vaccin* OR screening OR hospital* OR physician* OR utility OR utilities OR "discrete choice" OR "contingent valuation" OR "patient-reported" OR prom ) ) AND ( AFFILCOUNTRY ( turkey OR jordan OR leban* OR palestin* OR gaza OR "west bank" ) ) AND ( LIMIT-TO ( SUBJAREA , "MEDI" ) OR LIMIT-TO ( SUBJAREA , "SOCI" ) OR LIMIT-TO ( SUBJAREA , "PHAR" ) OR LIMIT-TO ( SUBJAREA , "DECI" ) OR LIMIT-TO ( SUBJAREA , "NURS" ) OR LIMIT-TO ( SUBJAREA , "HEAL" ) OR LIMIT-TO ( SUBJAREA , "ECON" ) OR LIMIT-TO ( SUBJAREA , "DENT" ) ) AND ( LIMIT-TO ( PUBYEAR , 2019 ) OR LIMIT-TO ( PUBYEAR , 2018 ) OR LIMIT-TO ( PUBYEAR , 2017 ) OR LIMIT-TO ( PUBYEAR , 2016 ) OR LIMIT-TO ( PUBYEAR , 2015 ) OR LIMIT-TO ( PUBYEAR , 2014 ) ) | 3823 |

1. NHSEED and HTA

(Turkey or Turkish or Lebanon or Lebanese or Jordan or Jordanian or Palestine or Palestinian or Gaza or West Bank) IN NHSEED, HTA WHERE LPD FROM 01/01/2014 TO 28/01/2019

18 hits

Appendix 3. Survey instrument

Welcome to this survey of capacity for **health economics** in the MENA region. The survey findings will inform the design and implementation of activities meant to strengthen capacity for health economics methods and applications in the region.

Please access the Participant Information Sheet below to find out more about the background of the survey and how your data will be analysed and protected. By completing the survey you agree for your answers to be analysed as described in the Participant Information Sheet.

[R4hc hesurvey pisheet v1.20](https://imperial.eu.qualtrics.com/CP/File.php?F=F_78aEDHCitG6tw7H)

If health economics is NOT relevant for your professional role, please exit the survey now by closing the browser window. However, please distribute the survey link to other colleagues for whom health economics is a relevant discipline.

Thank you in anticipation for contributing your answers.  

End of Block: Introduction

Start of Block: Institutional filter

| 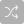 |
| --- |

Which of the following options best describes your current professional role?

 If you have more than one professional role, choose the one you spend most time in.

- Academic research
- Policy management, for example as a head of unit in a government agency
- Technical expert (non-academic), for example as a health financing specialist
- Healthcare management, for example as a hospital administrator
- Clinical activity, for example as a practicing physician or pharmacist
- Other ________________________________________________

End of Block: Institutional filter

Start of Block: Training and applied experience

Display This Question:

If Which of the following options best describes your current professional role?If you have more tha... != Policy management, for example as a head of unit in a government agency

And Which of the following options best describes your current professional role?If you have more tha... != Healthcare management, for example as a hospital administrator

| 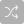 |
| --- |

Thinking about the past 5 years (since September 2014), choose the statements that apply to you for each of the following skills. 
 

Choose all statements that apply. For the purpose of this question, the following are NOT sufficient to be considered a 'course': one-day activities; individual study without an instructor; attending one-off lectures, conference presentations, seminars, webinars.

|  | No exposure to this topic | I completed an online course on this topic | I completed a face-to-face course on this topic | I have applied this skill in my work | I master this skill |
| --- | --- | --- | --- | --- | --- |
| Descriptive statistics, for example estimating averages, standard deviations, interquartile ranges |  |  |  |  |  |
| Simple regression analysis, for example logistic regression, ordinary least squares regression |  |  |  |  |  |
| Advanced regression analysis, for example hierarchical/multilevel/panel regression, generalized linear models |  |  |  |  |  |
| Systematic review of the literature |  |  |  |  |  |
| Meta-analysis |  |  |  |  |  |
| Design and collection of qualitative data, for example through interviews, ethnographic methods or focus groups |  |  |  |  |  |
| Analysis and interpretation of qualitative data, for example using framework analysis or thematic analysis |  |  |  |  |  |
| Social network analysis |  |  |  |  |  |
| Systems modelling, for example to analyse decision-making or program implementation |  |  |  |  |  |
| Decision analytic modelling, for example using decision trees, Markov models, discrete event simulation |  |  |  |  |  |
| Budget impact analysis |  |  |  |  |  |

Display This Question:

If Which of the following options best describes your current professional role?If you have more tha... != Policy management, for example as a head of unit in a government agency

And Which of the following options best describes your current professional role?If you have more tha... != Healthcare management, for example as a hospital administrator

| 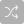 |
| --- |

Thinking about the past 5 years (since September 2014), choose the statements that apply to you for each of the following topics. Choose all statements that apply. For the purpose of this question, the following are NOT sufficient to be considered a 'course': one-day activities; individual study without an instructor; attending one-off lectures, conference presentations, seminars, webinars.

|  | No exposure to this topic | I completed an online course on this topic | I completed a face-to-face course on this topic | I have worked on this topic | I am an expert on this topic |
| --- | --- | --- | --- | --- | --- |
| **Economic evaluation** of health interventions, for example using cost-effectiveness analysis or cost benefit analysis |  |  |  |  |  |
| Measuring the **costs of health interventions**, for example through microcosting or activity-based costing |  |  |  |  |  |
| Measuring the **economic burden of disease**, for example through cost-of-illness studies or macroeconomic modelling |  |  |  |  |  |
| Measuring **health equity**, for example estimating the incidence of catastrophic health expenditure, benefit-incidence analysis, extended cost-effectiveness analysis |  |  |  |  |  |
| Measuring the **efficiency of health systems or health service providers**, for example using data envelopment analysis or stochastic frontier analysis |  |  |  |  |  |
| Measuring the **preferences** of health workers or patients, for example using discrete choice experiments, contingent valuation |  |  |  |  |  |
| Measuring **health utilities and health-related quality of life**, for example collecting and analysing individual-level data using EQ-5D or SF-6D instruments |  |  |  |  |  |
| **Formal policy analysis**, for example using the framework "Problem identification-Evidence gathering-Solution analysis-Policy recommendation" |  |  |  |  |  |
| **Political economy analysis** – national, sector-based, or problem-based |  |  |  |  |  |
| **Quasi-experimental methods**, for example interrupted time series, difference-in-difference, regression discontinuity design, synthetic control |  |  |  |  |  |

Display This Question:

If Which of the following options best describes your current professional role?If you have more tha... != Policy management, for example as a head of unit in a government agency

And Which of the following options best describes your current professional role?If you have more tha... != Healthcare management, for example as a hospital administrator

For the above topics that you have worked on, can you give a brief example of your applied experience and any challenges you faced?

 You can include references to published articles/reports, if available.
  
For example: I conducted a political economy analysis of the cancer care sector in my country and the main challenge I faced was the lack of political economy analysis experts to discuss and validate my findings with.

________________________________________________________________

Display This Question:

If Which of the following options best describes your current professional role?If you have more tha... = Policy management, for example as a head of unit in a government agency

Or Which of the following options best describes your current professional role?If you have more tha... = Healthcare management, for example as a hospital administrator

| 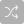 |
| --- |

Thinking about the past 5 years (since September 2014), choose the statements that apply to you for each of the following topics. 
Choose all statements that apply. For the purpose of this question, the following are NOT sufficient to be considered a 'course': one-day activities; self-guided individual study without an instructor; attending lectures, conference presentations, seminars, webinars.

|  | No exposure to this topic | I completed a face-to-face course on this topic | I completed an online course on this topic | I have worked on this topic | I am an expert on this topic |
| --- | --- | --- | --- | --- | --- |
| Identifying and appraising the available evidence for a given research question |  |  |  |  |  |
| Economic evaluation of health interventions, for example using cost-effectiveness analysis |  |  |  |  |  |
| Health technology assessment |  |  |  |  |  |
| Costing health benefit packages |  |  |  |  |  |
| Designing and reviewing health benefit packages |  |  |  |  |  |
| Designing and reviewing provider payment mechanisms |  |  |  |  |  |
| Equity analysis |  |  |  |  |  |
| Analytical tools for navigating the politics of health sector reform |  |  |  |  |  |
| Approaches for translating knowledge to policy and practice |  |  |  |  |  |

Display This Question:

If Which of the following options best describes your current professional role?If you have more tha... = Policy management, for example as a head of unit in a government agency

Or Which of the following options best describes your current professional role?If you have more tha... = Healthcare management, for example as a hospital administrator

For the above topics above that you have worked on, can you give a brief example of your applied experience and any challenges you faced?
 
You can include references to published articles/reports if available.
 
For example: I supervised/conducted the revision of the benefit package for paediatric oncology in my country and the main challenge was to obtain reliable service utilisation data from healthcare providers.

________________________________________________________________

End of Block: Training and applied experience

Start of Block: Interest in future development

Display This Question:

If Which of the following options best describes your current professional role?If you have more tha... != Policy management, for example as a head of unit in a government agency

And Which of the following options best describes your current professional role?If you have more tha... != Healthcare management, for example as a hospital administrator

| 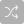 |
| --- |

Which of the following research skills do you consider most important for you to develop in the future? 
 
Order (drag and drop) the following options from the most important (1) to the least important (12).

______ Descriptive statistics, for example estimating averages, standard deviations, interquartile ranges

______ Simple regression analysis, for example logistic regression, ordinary least squares regression

______ Advanced regression analysis, for example hierarchical/multilevel/panel regression, generalized linear models

______ Systematic review of the literature

______ Meta-analysis

______ Design and collection of qualitative data, for example through interviews, ethnographic methods, focus groups

______ Analysis and interpretation of qualitative data, for example using framework analysis, thematic analysis

______ Social network analysis

______ Systems modelling, for example to analyse decision-making

______ Decision analytic modelling, for example using decision trees, Markov models, discrete event simulation

______ Budget impact analysis

______ Other

Display This Question:

If Which of the following options best describes your current professional role?If you have more tha... != Policy management, for example as a head of unit in a government agency

And Which of the following options best describes your current professional role?If you have more tha... != Healthcare management, for example as a hospital administrator

| 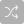 |
| --- |

Which of the following health economics topics do you consider most important for you to develop in the future?

 Order (drag and drop) the following options from the most important (1) to the least important (11).

______ **Economic evaluation** of health interventions, for example using cost-effectiveness analysis, cost benefit analysis

______ Measuring the **costs of health interventions**, for example through microcosting, activity-based costing

______ Measuring the **economic burden of disease**, for example through cost-of-illness studies, macroeconomic modelling

______ Measuring **health equity**, for example estimating the incidence of catastrophic health expenditure, benefit-incidence analysis, extended cost-effectiveness analysis

______ Measuring the **efficiency of health systems or health service providers**, for example using data envelopment analysis, stochastic frontier analysis

______ Measuring the **preferences** of health workers or patients, for example using discrete choice experiments, contingent valuation

______ Measuring **health utilities and health-related quality of life**, for example collecting and analysing individual-level data using EQ-5D or SF-6D instruments

______ **Formal policy analysis**, for example using the framework "Problem identification-Evidence gathering-Solution analysis-Policy recommendation"

______ **Political economy analysis** – national, sector-based, problem-based

______ **Quasi-experimental methods**, for example interrupted time series, difference-in-difference, regression discontinuity design, synthetic control

______ Other

Display This Question:

If Which of the following options best describes your current professional role?If you have more tha... = Policy management, for example as a head of unit in a government agency

Or Which of the following options best describes your current professional role?If you have more tha... = Healthcare management, for example as a hospital administrator

| 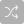 |
| --- |

Which of the following topics do you consider most important for you to develop in the future?

Order (drag and drop) the following options from the most important (1) to the least important (10).

______ Identifying and appraising the available evidence for a given research question

______ Economic evaluation of health interventions, for example using cost-effectiveness analysis

______ Health technology assessment

______ Costing health benefit packages

______ Designing and reviewing health benefit packages

______ Designing and reviewing provider payment mechanisms

______ Equity analysis

______ Analytical tools for navigating the politics of health sector reform

______ Approaches for translating knowledge to policy and practice

______ Other

| 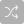 |
| --- |

For the top 3 topics you ranked above, what would be your preferred learning style? 
 
Order (drag and drop) the following options from the most preferred (1) to the least preferred (8).

______ Online course with graded assessment(s) and certificate of completion

______ Face-to-face course (2-5 days)

______ Learn by doing, for example working on a practical task with minimal supervision

______ Online course without graded assessment(s)

______ Direct mentoring from an experienced professional

______ Master-level course offered by an academic institution

______ Peer-to-peer learning, for example interaction with professionals who share the same challenges as you

______ Other

Considering the same top 3 topics, could you give a brief example of how you would like to use them in your work?
 
For example: conduct a cost-effectiveness analysis of ... in order to inform ... .

________________________________________________________________

End of Block: Interest in future development

Start of Block: Respondent background

Which country do you work in?

________________________________________________________________

| 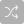 |
| --- |

What type of organisation do you work for?

 If you work for more than one organisation, choose the one you spend most time with.

- University or research institute
- Non-governmental organisation (NGO)
- Healthcare provider, for example a hospital
- Government agency
- International organisation
- Private sector, for example consultancy or pharmaceutical industry
- Other ________________________________________________

(Optional) Your institution and department.
 
For example: Ministry of Health, Non-communicable disease unit.

________________________________________________________________

Highest level of education completed.

- Undergraduate degree (for example: Medicine, Dentistry, Economics)
- Master (for example: MA, MSc, MPH)
- Doctorate (for example: PhD, DrPH)
- Other ________________________________________________

Years since the highest level of education completed.

- Less than 5 years
- Between 5 years and 10 years
- More than 10 years

(Optional) Type your email address below if you wish to 1) receive information about future health economics-related activities in the R4HC project; and 2) receive a copy of this survey's findings when they become available.
 
Your email address will be delinked from your responses before analysis, will be kept CONFIDENTIAL, will not be shared with any other party and will not be used for any other purposes than specified above.

________________________________________________________________

End of Block: Respondent background

Start of Block: Respondent feedback

Is there anything else you want to share about your professional experience, interests or this survey?

________________________________________________________________

End of Block: Respondent feedback

Appendix 4. Detailed results of the online survey

Figure A4.1 Respondents’ profile by jurisdiction and professional role (n=80)


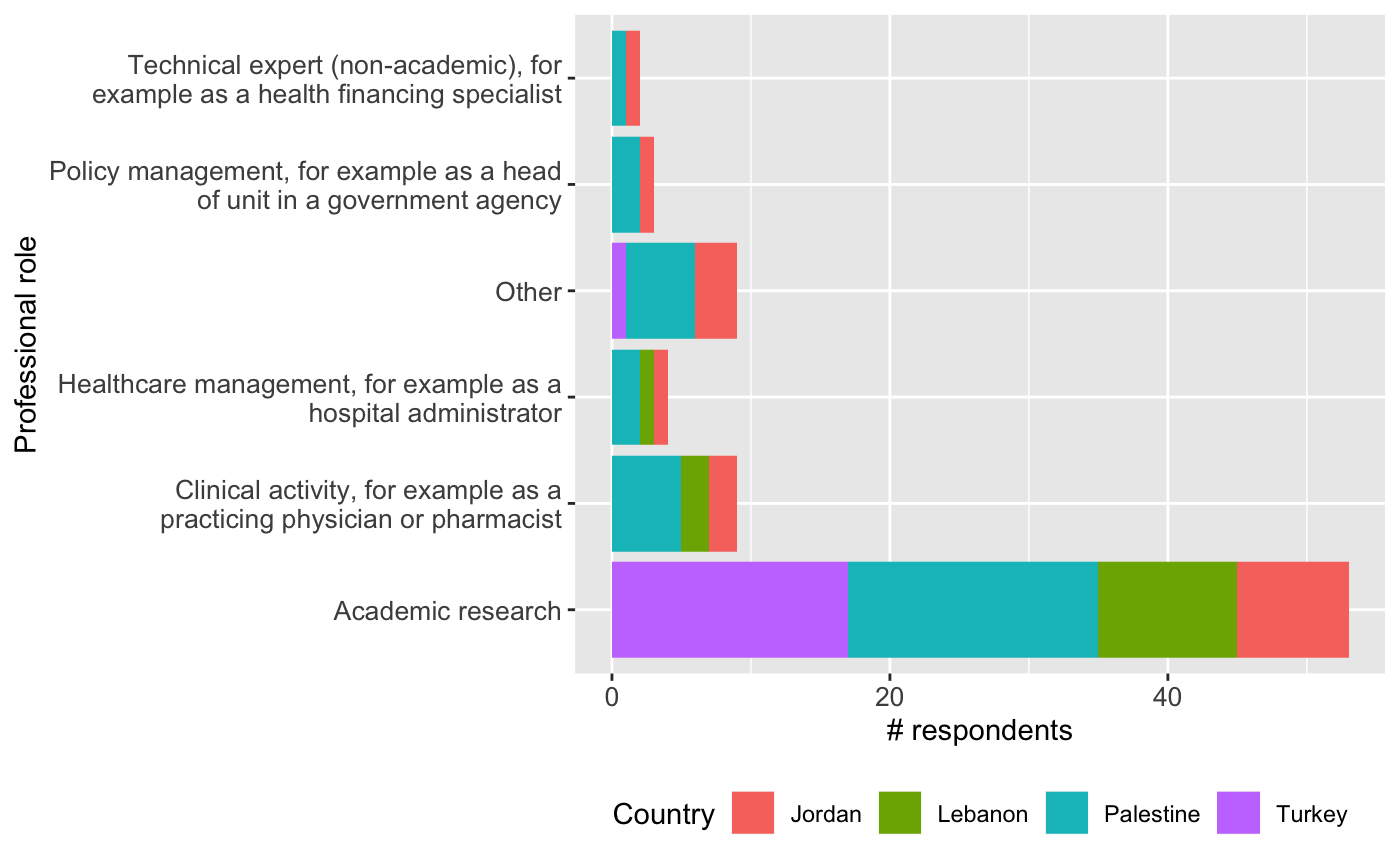


Figure A4.2 Respondents’ profile by jurisdiction and type of employer (n=80)


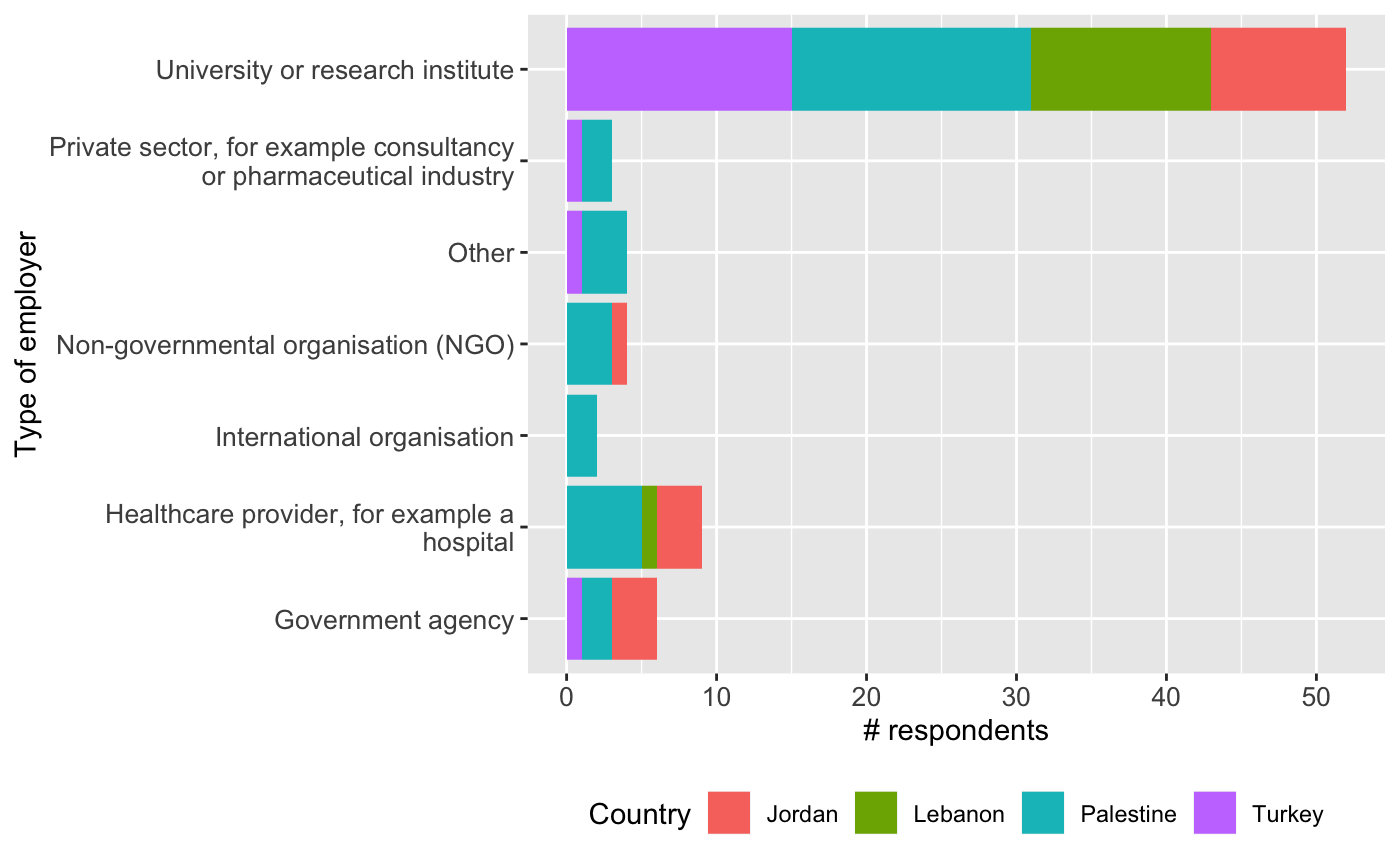


Figure A4.3 Respondents’ profile by jurisdiction and highest degree completed (n=80)


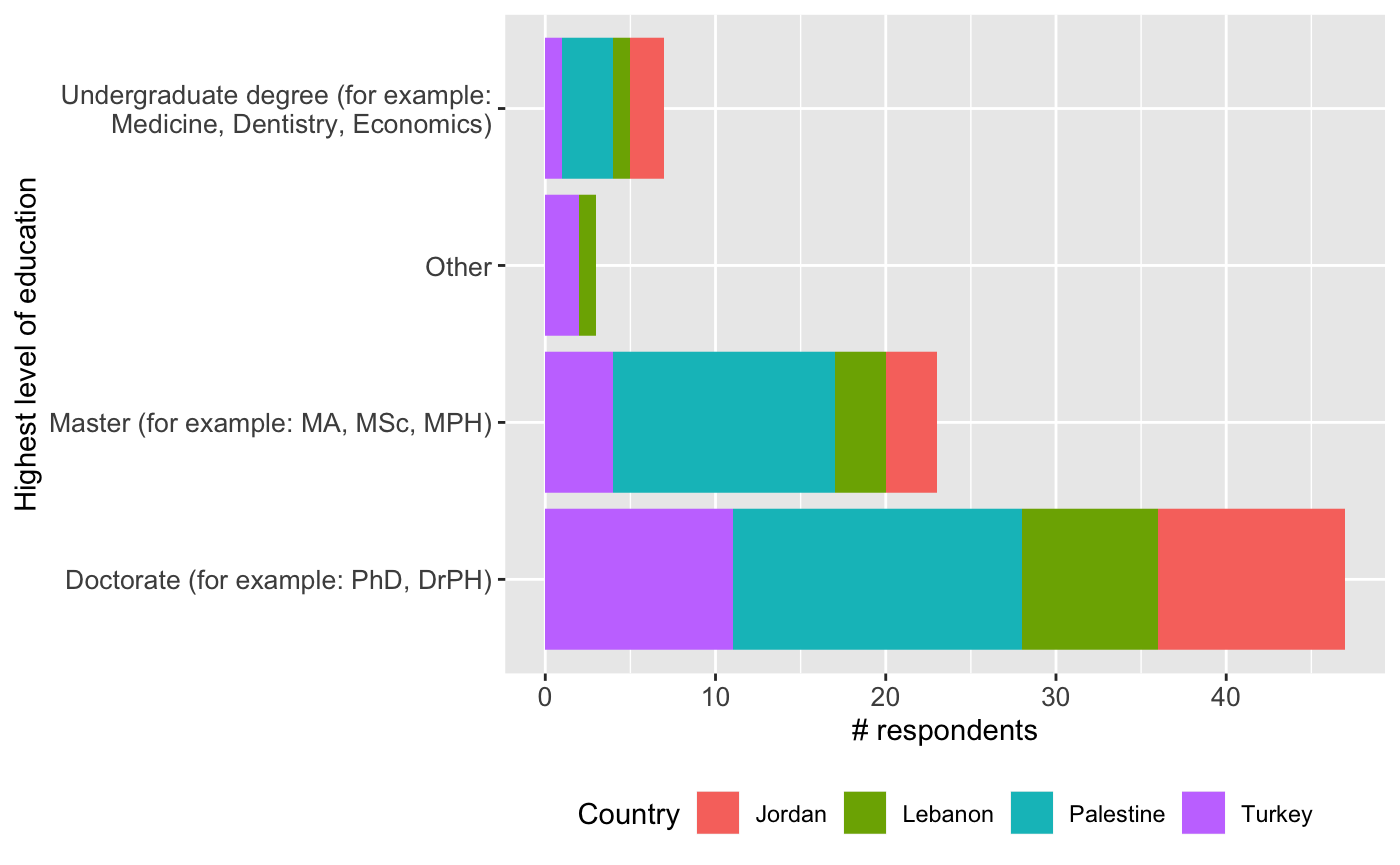


Figure A4.4 Respondents’ profile by jurisdiction and length of professional experience (n=80)


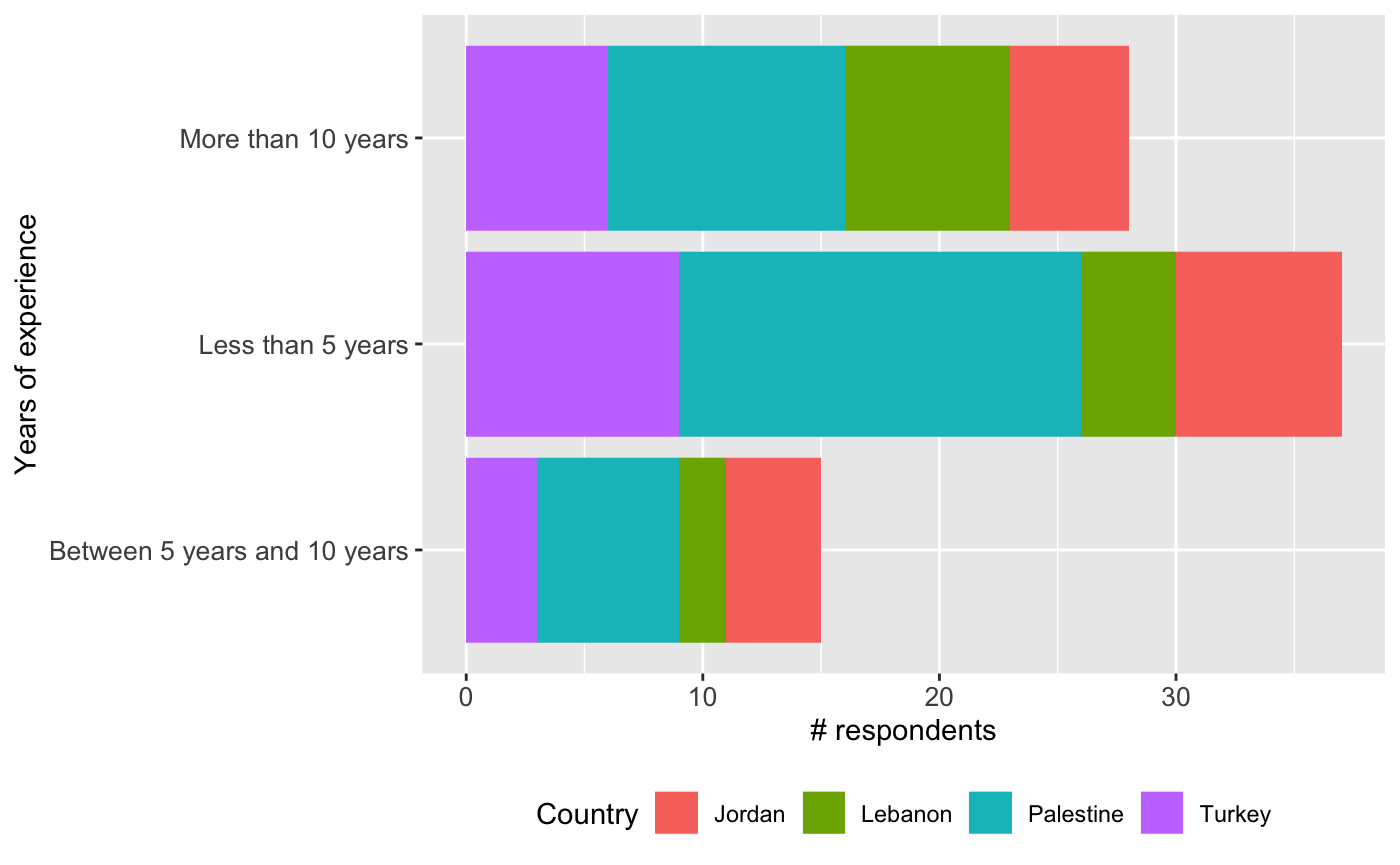


Figure A4.5 Previous exposure to generic research skills, by jurisdiction (n=73)


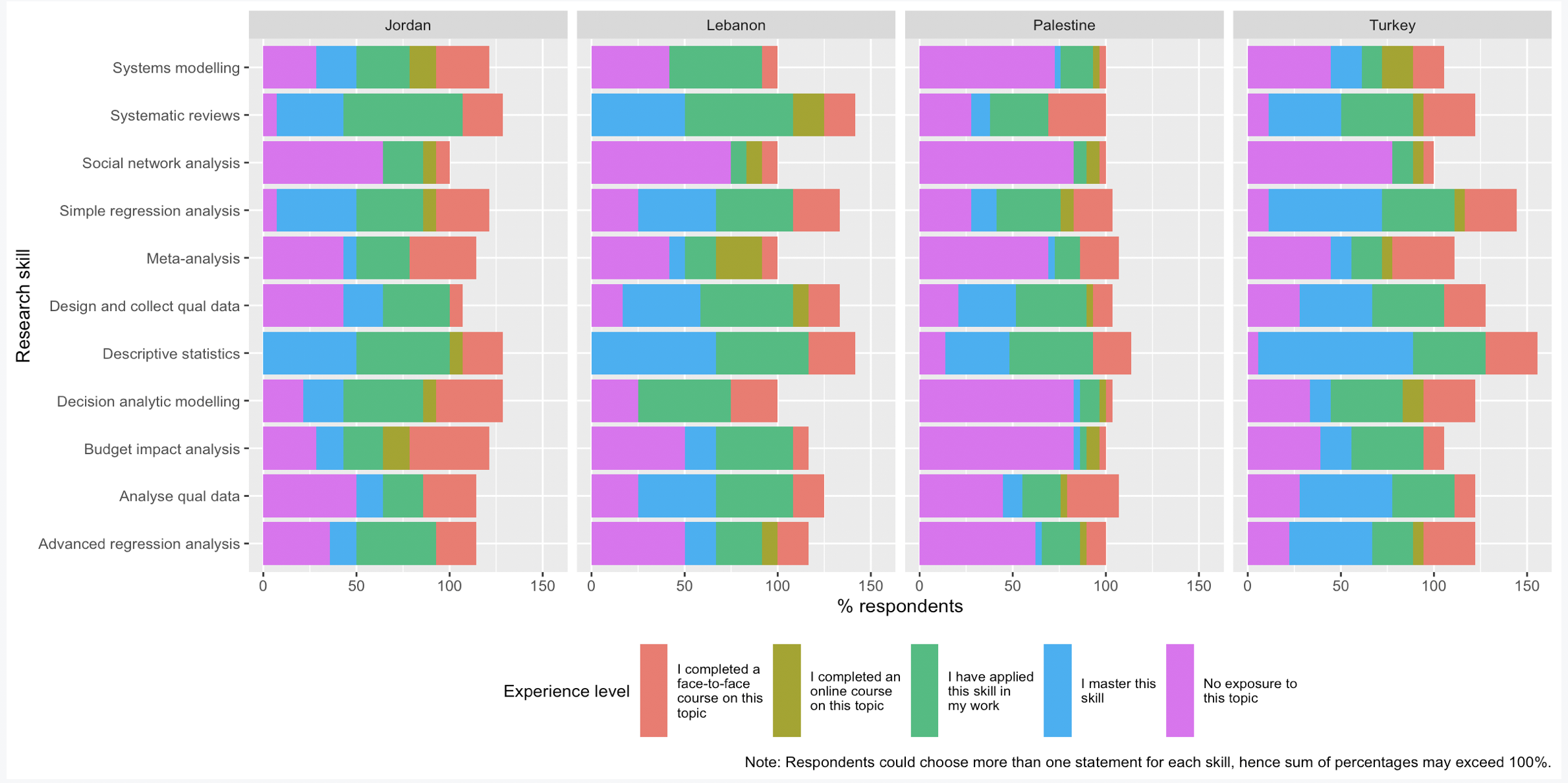


Figure A4.6 Previous exposure to health economics topics, by jurisdiction (n=73)


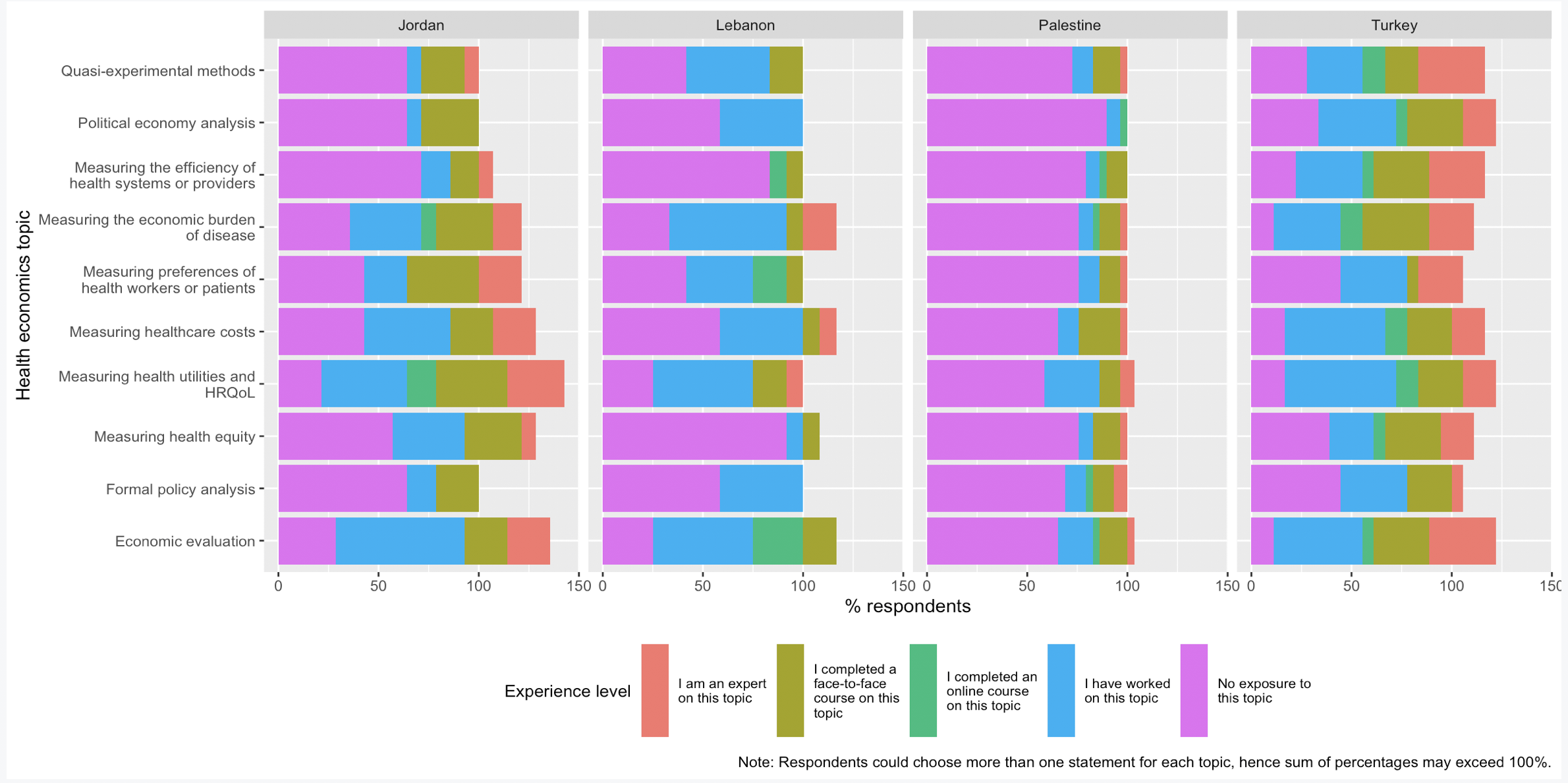


Figure A4.7 Priorities for future development in generic research skills, by jurisdiction (n=73)


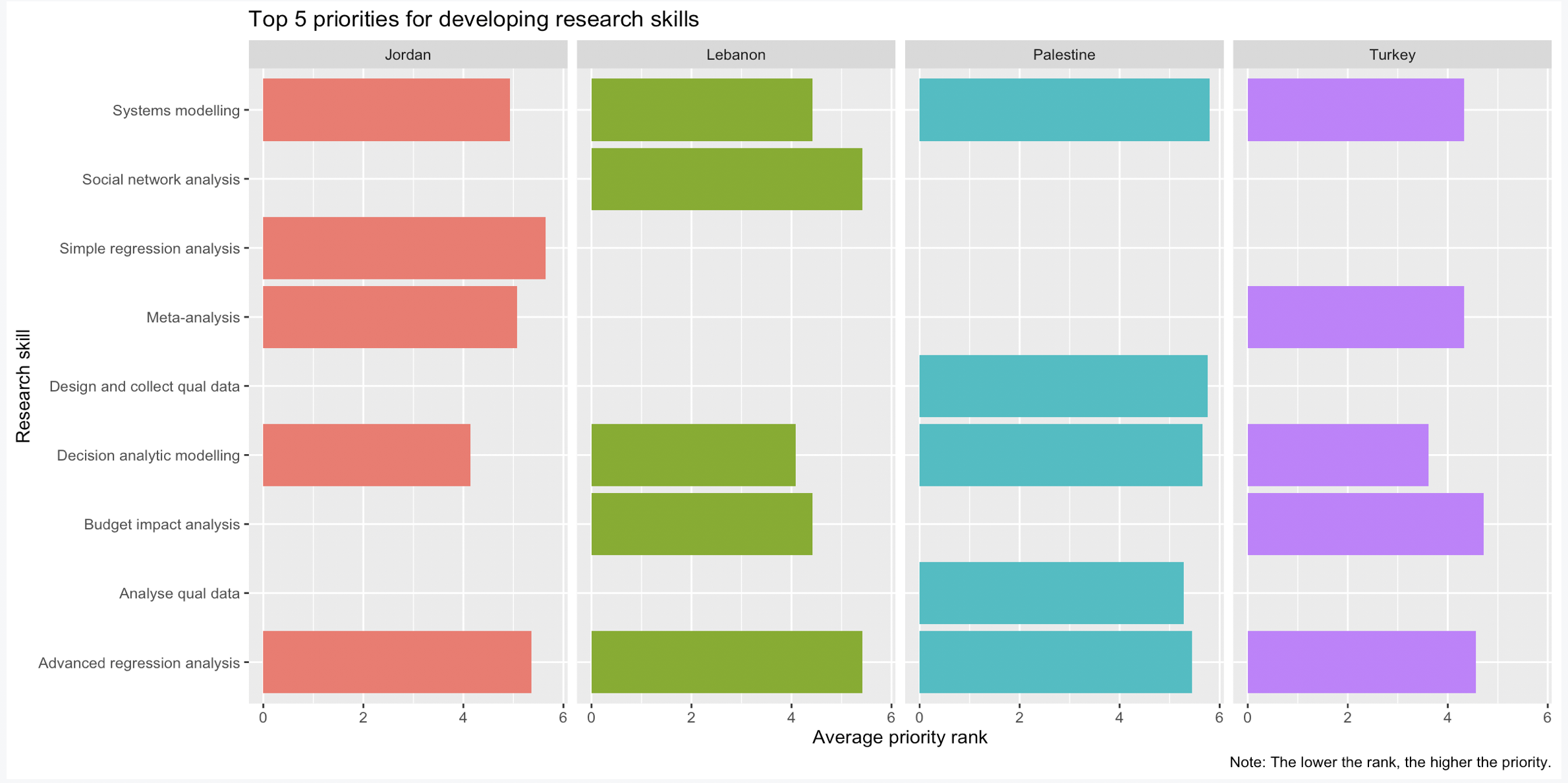


Figure A4.8 Priorities for future development in health economics topics, by jurisdiction (n=73)


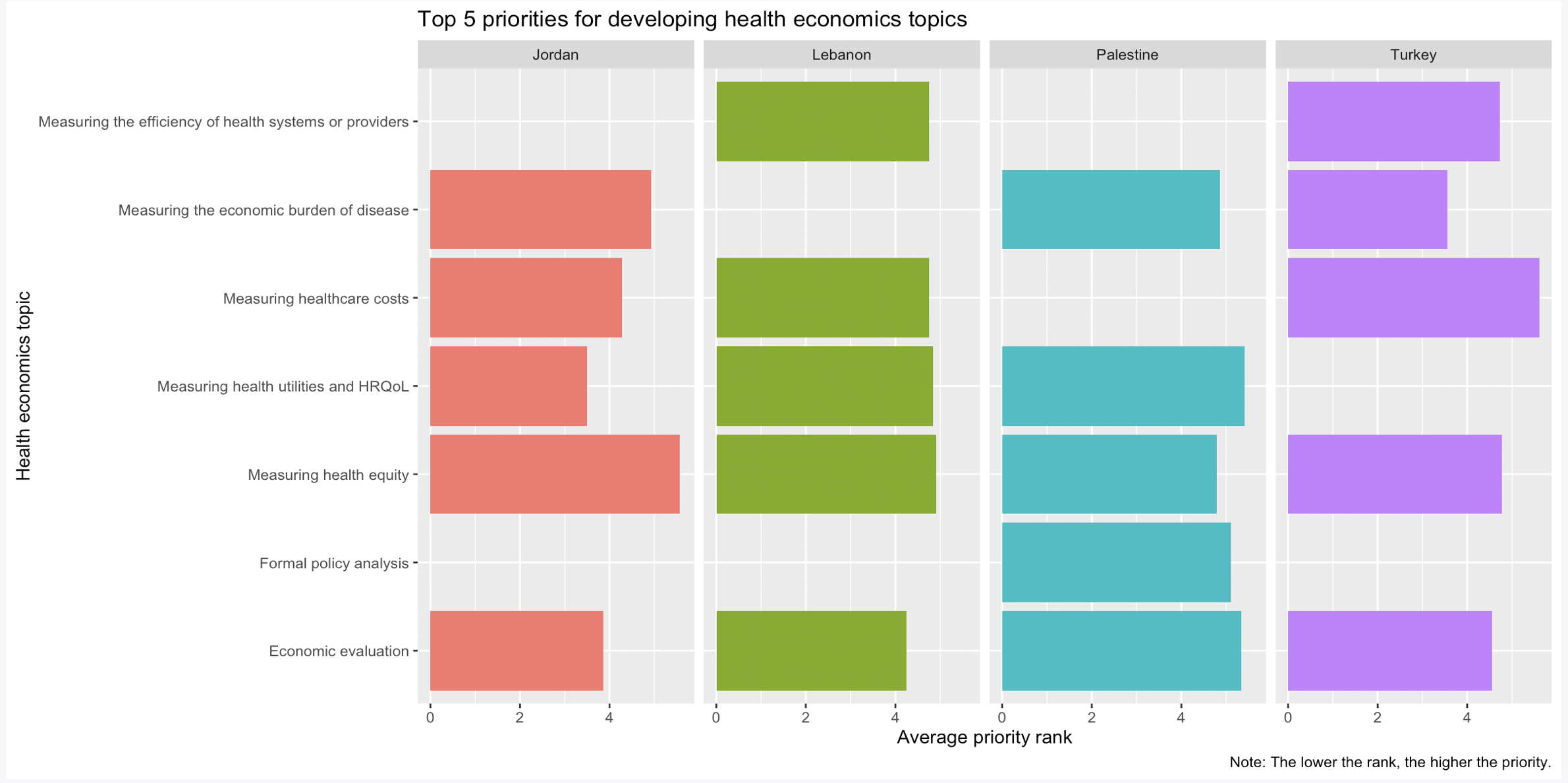


Figure A4.9 Priorities for future development among policy managers and healthcare administrators (n=7)


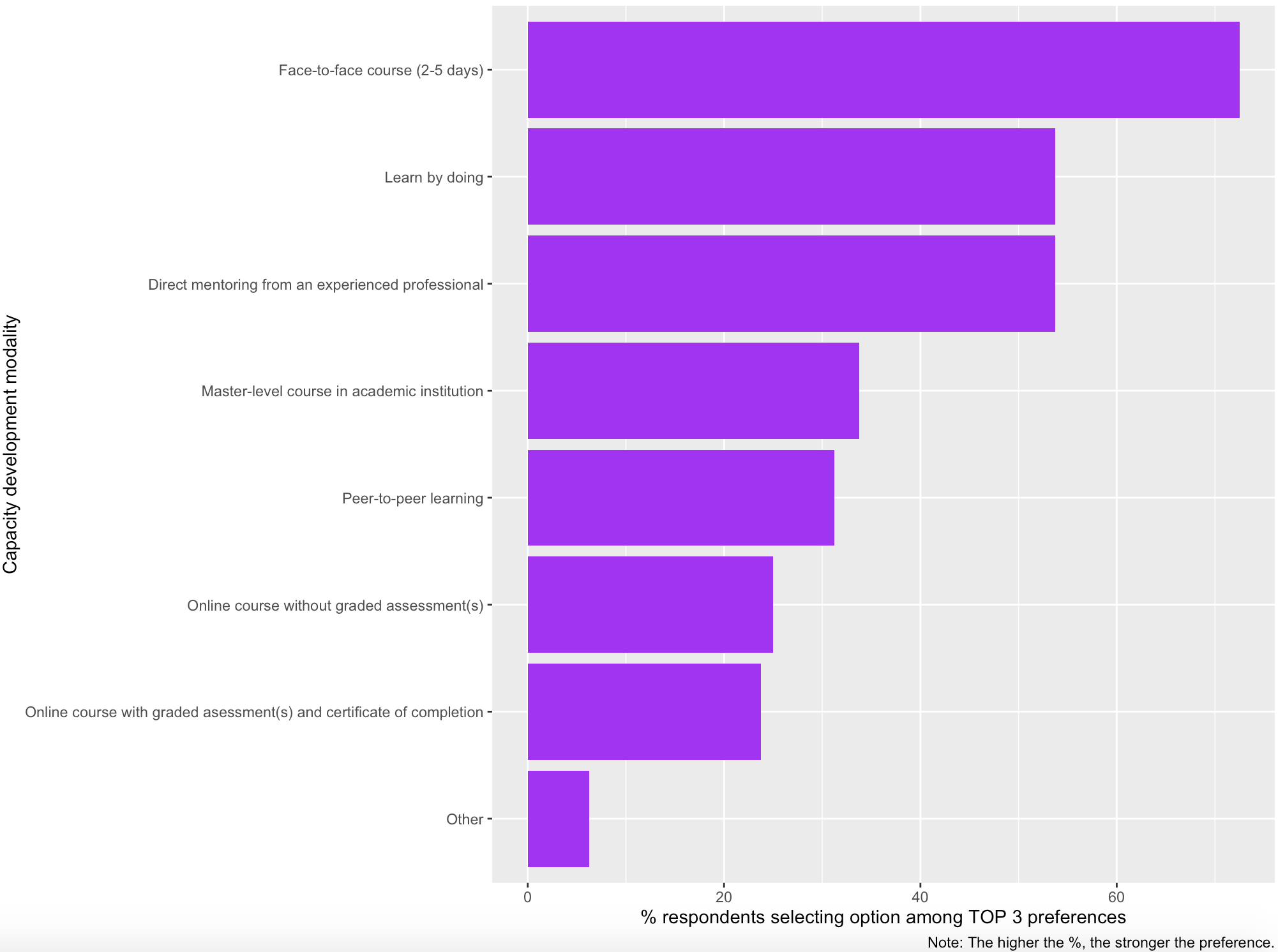


Figure A4.10 Preferences for learning styles, by jurisdiction (n=80)


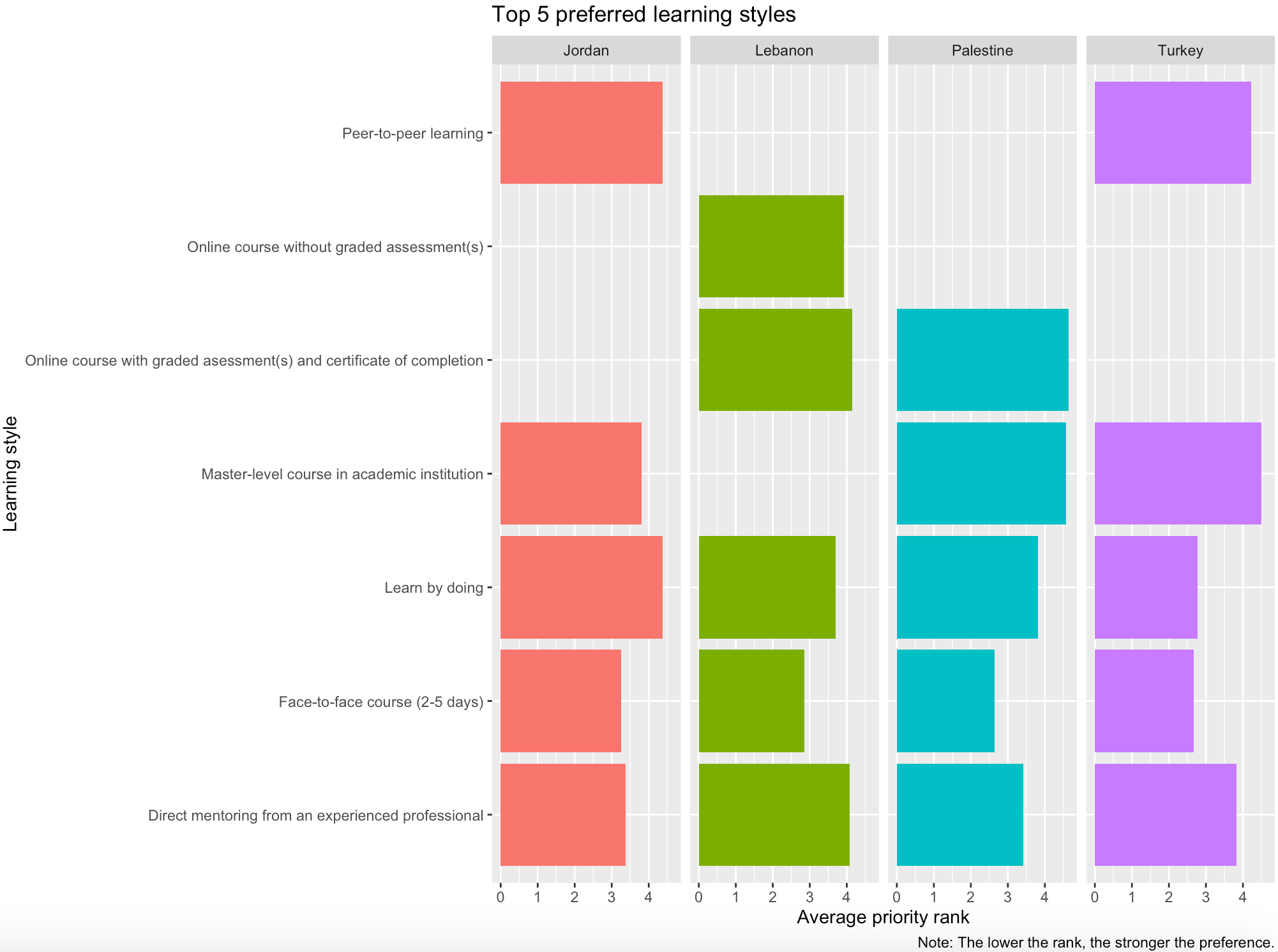

Supplement: Supplementary file 1 — Additional file 1. Supplementary information. [file 12961_2020_586_MOESM1_ESM.docx]
